# Supplementary material for: Emergence of novel cephalopod gene regulation and expression through large-scale genome reorganization
Source: Nat Commun. 2022 Apr 21;13:2172. doi: 10.1038/s41467-022-29694-7 (PMC9023564; doi:10.1038/s41467-022-29694-7)
Supplement: Supplementary file 1 — Supplementary Information [file 41467_2022_29694_MOESM1_ESM.pdf]

Supplementary Notes

Schmidbaur *et al.*, Emergence of novel cephalopod gene regulation and expression through large-scale genome reorganization

1. Animal collection and fixation
2. Genome scaffolding
    2.1. *In situ* Hi-C preparation for scaffold assembly
    2.2. Genome scaffolding
    2.3. Annotation liftover
    2.4. Hi-C mapping and contact matrices
3. Synteny analysis
    3.1. Orthology, synteny clustering and randomization of syntenic blocks
    3.2. Circos plot
    3.3. Density analysis of micro-syntenies, microsyteny distribution and karyoplots
    3.4. GO term enrichment analysis
4. Chromatin conformation analysis
    4.1. TAD prediction
    4.2. Synteny and TAD composition
    4.3. TAD averaging
    4.4. Motif finding (TAD boundaries)
    4.5. Protein analysis
5. 3D modelling and SASA analysis
6. Neighbour-joining method for TAD syntenic consistency profiling
7. Co-expression analysis and GO term enrichment
8. Annotation of putative CNEs
9. Chromatin accessibility assay with ATAC-seq
    9.1. ATAC-seq library preparation
    9.2. ATAC-seq - Mapping, quality control and peak calling
    9.3. Motif finding ATAC-seq, repeat annotation of ATAC-peaks
10. Orthologous expression comparison to scallop
11. *In situ* hybridization

Supplementary Note 1. Animal collection and fixation

Euprymna scolopes eggs were obtained from cultures from Spencer Nyholm (Department of Molecular and Cell Biology, University of Connecticut, Storrs, USA), Jamie S. Foster (University of Florida, Space Life Science Lab, Merritt Island, USA) and the Marine Biological Laboratory's Cephalopod Breeding Initiative (Woods Hole, Massachusetts, USA, aff. University of Chicago) and kept at Vienna Schoenbrunn Zoo.

Animals were collected at different time points and embryos were removed from egg cases and jelly layers. Older embryos and hatchlings were anesthetized in 4 % EtOH in seawater or 4 % EtOH and $MgCl_2$ (2M solution added slowly to seawater)[1–4] prior to fixation. For ATAC-seq and Hi-C animals were used immediately as described in the corresponding sections. For in-situ hybridization, animals were fixed in 4 % paraformaldehyde in marine PBS (http://glowingsquid.org/files/Protocol_File/FixAndStainJuvenileLightOrgans.pdf) for 6 h or overnight. Animals were washed 3x in PBS and stored in Hybe buffer (0.1 g heparin, 2.5 g yeast RNA, 0.25 g acetylated Bovine Serum Albumin, 125 ml 20x SSC (pH 4.5), 250 ml Formamide (50%), 50 ml 10% SDS, filled up to 500 ml with water) at -20°C. Animals for RNA-seq were all collected from the same clutch. Five embryos were used per replicate and three replicates per stage were sampled. As much of the seawater as possible was removed, 50µl of TRIzol® Reagent (Life Technologies, Carlsbad, California, United States) was added and embryos were flash frozen in liquid nitrogen and kept at -80°C until library preparation.

Supplementary Note 2. Genome assembly and analysis

2.1 *In situ* Hi-C library preparation

All Hi-C libraries from this paper were generated as described in [5–7], with modifications as described below. After removing the chorion and yolk sac, embryos of stage ~27 were dissociated in Liberase in PBS (1:100 dilution Unit of Liberase ™, for 15 min at RT). The cells were collected by centrifugation (380 x g for 10 min at 4°C), and resuspended into 9 ml of 10 % FBS/PBS. The cells were fixed with formaldehyde (1 % f. c. (Merck (Burlington, Massachusetts, United States))) for 10 minutes at room temperature. Fixation was stopped by adding ice-cold glycine (125 mM f. c. (Sigma-Andrich (St. Louis, Missouri, United States))). Fixed cells were collected by centrifugation (380 x g for 10 min at 4°C), and washed with ice-cold PBS twice (380 x g for 10 min at 4°C). Cells at a concentration of 1 × 10^6 per ml were collected and incubated in

ice-cold lysis buffer on ice (10 mM Tris–HCl pH 8, 10 mM NaCl, 0.2% Igepal CA-630, 1 % Triton-X100, protease inhibitor cocktail EDTA free). After centrifugation to pellet the cell nuclei (380 x g for 10 min at 4°C) were washed once with 1.25x NEBuffer2 (New England Biolabs (Ipswich, Massachusetts, United States)). The nuclei were resuspended in 1.25x NEBuffer2. SDS (0.6 % f. c.) was added, and the mixture was incubated with agitation (950 rpm for 2 h at 37°C). Triton X-100 (3.3 % f. c.) was added to quench the SDS, and the nuclei were incubated with agitation (950 rpm for 2 h at 37°C).

Restriction digestion with HindIII (from NEB; 2,000 U per 0.25 million cells) was performed overnight with agitation (950 rpm at 37°C). After chromatin digestion, the restriction sites were filled with biotin-14-dATP (Thermo Fisher (Waltham, Massachusetts, United States)), dCTP, dGTP, and dTTP, with Klenow (50 U per 0.5 million cells) for 1 h at 37°C with repeated agitation (700 rpm 10 sec and rest 30 sec for 1 h in a thermal cycler).

Ligation was performed overnight at 18°C (2000 U of T4 DNA ligase). After ligation, crosslinking was reversed by proteinase K treatment overnight at 65°C. An additional proteinase K incubation at 65°C for 2 h was followed with RNase A treatment and two sequential phenol/chloroform extractions. After DNA precipitation, the DNA was spun down (centrifugation with max-speed for 30 min at 4°C). The pellets were resuspended in 20 µl TE and the DNA concentration was determined using Qubit 2 device (Thermo Fisher).

20 µg of Biotinylated DNA was used for the library preparation. The biotin from non-ligated fragment ends was removed with T4 DNA polymerase (NEB) for 30 min at 37°C and EDTA was added to stop the reaction (10 mM f. c.). DNA was sonicated using the Covaris system to generate DNA fragments with a size peak around 400 bp (Covaris S2 settings: duty factor: 10%; peak incident power: 5; time: 60 sec). After end repair with T4 DNA polymerase, Klenow Large fragment and T4 DNA polynucleotide kinase in the presence of dNTPs in T4 DNA ligation buffer (for 30 min at room temperature), the DNA was purified (QIAGEN mini purification kit (QIAGEN (Hilden, Germany))).

A double-size selection using DNA purification beads was performed and the size selected DNA was eluted in Elution buffer (QIAGEN). Biotinylated ligation products were isolated using pre-washed MyOne Streptavidin C1 Dynabeads (Life Technologies) on a magnet stand in binding buffer (5 mM Tris pH8, 0.5 mM EDTA, 1 M NaCl) for 30 min at room temperature. dA-tailing was carried on beads: dATP was added with Klenow exo- (for 1h at 3°C, NEB), then the enzyme was heat-inactivated (20 min at 65°C). After two washes in binding buffer and one wash in T4 DNA ligation buffer, Illumina adapters were ligated onto Hi-C ligation products bound to streptavidin beads in T4 DNA ligase by slow rotation (for 2 h at room temperature). After

washing twice with wash buffer (5 mM Tris pH 8.0, 0.5 mM EDTA, 1 M NaCl, 0.05 % Tween-20) and then once with binding buffer, the DNA-bound beads were resuspended in a final volume of 20 µl 1x NEBBuffer2. Captured biotinylated Hi-C DNA was amplified by 9 cycles PCR amplification (with NEBNext® High-Fidelity 2X PCR Master Mix (NEB)). After PCR amplification, the Hi-C libraries were purified with DNA purification beads. Hi-C libraries were sequenced by paired-end 50 bp mode at VBCF NGS facility (Vienna BioCenter, Vienna, Austria).

2.2 Genome scaffolding

Genome scaffolding based on Hi-C data was done using Lachesis[8]. Scaffolds of the published *Euprymna scolopes* genome[9] were filtered to be at least 50k in size (changing the N50 from 3.5 MBp to 3.7 MBp) and used as draft de-novo assembly. Bam files aligned to the draft assembly of the two Hi-C datasets were used as Hi-C read input. Alignment was done as a step in the HiC-Pro[10] pipeline, thus HiC-Pro settings for bowtie[11] were used (see next section). `RE_SITE_SEQ` was speficied as AAGCTT. Other settings were: `CLUSTER_CONTIGS_WITH_CENS = -1`, `CLUSTER_MIN_RE_SITES = 25`, `CLUSTER_MAX_LINK_DENSITY = 2`, `CLUSTER_NONINFORMATIVE_RATIO = 3`, `CLUSTER_DRAW_HEATMAP = 0`, `CLUSTER_DRAW_DOTPLOT = 1`, `ORDER_MIN_N_RES_IN_TRUNK = 15`, `ORDER_MIN_N_RES_IN_SHREDS = 15`.

As the exact number of chromosomes is unknown for *E. scolopes* different numbers for `CLUSTER_N` (expected chromosome number) were tried (35, 40, 45, 46, 48, 50, 60), according to the number of other published cephalopod chromosomes. Then, the resulting heatmaps were checked for errors. Setting `CLUSTER_N` to 46 resulted in 48 well-defined clusters without noticeably misplaced contigs in the contact map, while lower or higher settings showed obvious errors in the clustering. Lachesis was able to assign 92.83% of all contigs to clusters (Supplementary Table 1).

Supplementary Table 1. Assembly statistics

N contigs:	3876
Total length:	5114188061
N50:	3723741
N clusters (derived):	48
N non-singleton clusters:	48
N orderings found:	48
Number of contigs in clusters:	3598 (92.83% of all contigs)
Length of contigs in clusters:	5070958786 (99.15% of all sequence length)
Number of contigs in orderings:	2189 (60.84% of all contigs in clusters, 56.48% of all contigs)
Length of contigs in orderings:	4821760194 (95.09% of all length in clusters, 94.28% of all sequence length)
Number of contigs in trunks:	988 (45.13% of contigs in orderings)
Length of contigs in trunks:	1656726814 (34.36% of length in orderings)
Fraction of contigs in orderings with high orientation quality:	2184 (99.77%), with length 4819879074 (99.96%)

2.3 Annotation lift-over

Genes were lifted over from the published annotation using an in-house script. The script uses the sizes of old and new scaffolds and the ordering files provided by Lachesis, which contain a list of all scaffolds assembled to a pseudo-chromosome in the assembled order. Between each scaffold 1001 bp are added as Ns.

2.4 Hi-C mapping and contact matrices

Two biological Hi-C replicates were mapped against the unmasked reference genome after filtering out scaffolds smaller than 50k, without trimming, using HiC-Pro[10]. Bowtie settings were: Global options: `--very-sensitive -L 30 --score-min L,-0.6,-0.2 --end-to-end --reorder. Local options= --very-sensitive -L 20 --score-min L,-0.6,-0.2 --end-to-end --reorder`. Other settings were: `Normalization maxiter = 100 filterlowcountpercent = 0.02 filterhighcountpercent = 0 EPS = 0.1`. This resulted in 106923369 valid interaction pairs (26696201FF, 26682284RR, 26435507RF, 27109377FR), 11294484 dangling end pairs, 3489522 relegation pairs, 704787 self cycle pairs, 0 single-end pairs and 168358 dumped pairs. The aligned bam files were then used for chromosome scaffolding with Lachesis as described above. After scaffolding the *E. scolopes* genome to 48 pseudo-chromosomes, the Hi-C reads were again aligned with HiC-Pro to the new assembly, using the same settings as above. This resulted in 106928662 valid interaction pairs (26702993FF, 26687521RR, RF 26451618, FR 27086530) 11294213 dangling end pairs, 3491065 relegation pairs, 704973 self cycle pairs, 0 single end pairs and 168420 dumped pairs.

Supplementary Note 3. Synteny analysis

3.1 Orthology, synteny clustering and randomization of syntenic blocks

Orthologous genes between 27 species (25 with genomic information, Supplementary Table 2) covering major metazoan lineages were identified using OrthoFinder[12,13]. When associated with a genome assembly, protein files were filtered by isoforms, retaining only the longest transcript per gene. These sequences were compared in all vs. all BLASTP ([14]version 2.10.0+) searches (e-value cutoff < 1e-3) and the results were further used for the reconstruction of orthogroups with OrthoFinder (version 2.3.7), using default parameters. The following protein files were included in the analysis:

Supplementary Table 2. Species used in the orthology

**Abbreviation**	**Species name**	**Assembly/Transcriptome**	**Source**
OCTBI	*Octopus bimaculoides*	PRJNA270931 https://metazoa.ensembl.org/Octopus_bimaculoides/Info/Index	ENSEMBL
CALMI	*Callistoctopus minor*	PRJNA421033 http://gigadb.org/dataset/100503	GIGA DB
EUPSC	*Euprymna scolopes*	New assembly Reference assembly: GCA_004765925.1 https://www.ncbi.nlm.nih.gov/assembly/GCA_004765925.1/	NCBI
IDIPA	*Idiosepius paradoxus*	Transcriptome data SAMN00152410 https://www.ncbi.nlm.nih.gov/biosample/SAMN00152410/	NCBI
DORPE	*Doryteuthis pealeii*	Transcriptome data SAMN00691532 https://www.ncbi.nlm.nih.gov/biosample/?term=SAMN00691532	NCBI
HELRO	*Helobdella robusta*	Helro1 http://metazoa.ensembl.org/Helobdella_robusta/Info/Index	ENSEMBL
LOTGI	*Lottia gigantea*	Lotgi1 http://metazoa.ensembl.org/ Lottia_gigantea/Info/ Index	ENSEMBL
CRAGI	*Crassostrea gigas*	oyster_v9 http://metazoa.ensembl.org/Cr	ENSEMBL

		assostrea_gigas/Info/Index	
APLCA	*Aplysia californica*	AplCal3.0 GCA_000002075.2 https://www.ncbi.nlm.nih.gov/assembly/GCF_000002075.1/	NCBI
MIZYE	*Mizuhopecten yessoensis*	GCA_002113885.2 https://www.ncbi.nlm.nih.gov/assembly/GCF_002113885.1/	NCBI
SCHMA	*Schistosoma mansoni*	ASM23792v2 http://metazoa.ensembl.org/Schistosoma_mansoni/Info/Index	ENSEMBL
CAPTE	*Capitella teleta*	Capitella_teleta_v1.0 http://metazoa.ensembl.org/Capitella_teleta/Info/Index	ENSEMBL
CAEEL	*Caenorhabditis elegans*	WBcel235 http://m.ensembl.org/Caenorhabditis_elegans/Info/Annotation	ENSEMBL
DROME	*Drosophila melanogaster*	BDGP6.28 http://www.ensembl.org/Drosophila_melanogaster/Info/Index	ENSEMBL
STEMI	*Stegodyphus mimosarum*	Stegodyphus_mimosarum_v1 (https://metazoa.ensembl.org/Stegodyphus_mimosarum/Info/Index	ENSEMBL
TRICA	*Tribolium castaneum*	Tcas5.2 http://metazoa.ensembl.org/Tribolium_castaneum/Info/Index	ENSEMBL
ADIVA	*Adineta vaga*	AMS_PRJEB1171_v1 https://metazoa.ensembl.org/Adineta_vaga/Info/Index	ENSEMBL
STRPU	*Strongylocentrotus purpuratus*	Spur_3.1 https://www.ncbi.nlm.nih.gov/assembly/GCF_000002235.3/	NCBI

ACAPL	*Acanthaster planci*	GCA_001949145.1 OLI-Apl_1.0 https://www.ncbi.nlm.nih.gov/assembly/GCF_001949145.1/	NCBI
SACKO	*Saccoglossus kowalevskii*	GCA_000003605.1 https://www.ncbi.nlm.nih.gov/assembly/GCF_000003605.2	NCBI
CIOIN	*Ciona intestinalis*	GCA_000224145.2 https://www.ncbi.nlm.nih.gov/assembly/GCF_000224145.3	NCBI
MOUSE	*Mus musculus*	GRCm38.p6 (GCA_000001635.8) https://www.ncbi.nlm.nih.gov/assembly/GCF_000001635.26/	NCBI
BRAFL	*Branchiostoma floridae*	GCA_000003815.1 Version 2 https://www.ncbi.nlm.nih.gov/assembly/GCF_000003815.1/#/st	NCBI
HUMAN	*Homo sapiens*	GRCh37.p13 (GCA_000001405.1) https://grch37.ensembl.org/Homo_sapiens/Info/Index	ENSEMBL
NEMVE	*Nematostella vectensis*	ASM20922v1 https://metazoa.ensembl.org/Nematostella_vectensis/Info/Index	ENSEMBL
AMPQU	*Amphimedon queenslandica*	Aqu1 https://metazoa.ensembl.org/Amphimedon_queenslandica/Info/Index	ENSEMBL
MNELE	*Mnemiopsis leidyi*	MneLei_Aug2011 http://metazoa.ensembl.org/Mnemiopsis_leidyi/Info/Index	ENSEMBL

Microsyntenic blocks were identified as previously described in [15,16]. Blocks were constrained to consist of least three genes, with no more than five intervening genes. The maximum number of paralogues was set to 100. Further filtering settings: minimum overlap 0.3 (at least 30% of orthogroups must overlap in any given syntenic loci pair) and minimum species overlap 0.5 (orthogroup present in at least half of the species in the block).

Idiosepius paradoxus, *Doryteuthis pealeii* and *Acanthaster plancii* which were used for the ortholog assignment were excluded from further analysis, thus 24 species were used for clustering. Using the resulting clustering file, we defined **metazoan** syntenies to be present in at least seven species out of the 24 initial species. **Cephalopod-specific** synteny was defined to be present in at least two cephalopods, but none of the other species. Then clusters present in *E. scolopes* were used for further analysis, thus all clusters that are present in *E. scolopes* and six other species (275 clusters) were used for the analysis of ancestral, metazoan clusters in *E. scolopes* and all clusters present in *E. scolopes* and shared with at least one other cephalopod (505 clusters, of which five are paralogous clusters) were used for the analysis of novel, cephalopod-specific clusters.

Counting of novel microsyntenies

Novel microsyntenies emerging at specific branches of the taxon tree (Supplementary Figure 2) were filtered. Each microsyntenic cluster had to contain at least wto species out of a list defining the group (*e.g.*, cephalopods - *Octopus bimaculoides*, *Callistoctopus minor*, *Euprymna scolopes*) and no species that was not in the list.

Those pre-filtered microsyntenic clusters were then used to calculate the number of novel micro-syntenic clusters for each branch. Paralogous clusters were excluded by using the clustering file with information of all connections between species in each syntenic block and only counting each species once (even if they have more than one connection to a syntenic block). Specific filtering parameters are listed in Supplementary Table 3.

Supplementary Table 3. Microsyntenic block age estimation

Taxon	Filtering parameters
**Spiralia**:	At least one species out of each spiralian group (Mollusca, Annelida and (Platyhelminthes + Rotifera)) or at least one species in Mollusca and one species in Annelida
Mollusca:	At least one species out of all three molluscan groups (Bivalvia, Gastropoda, Cephalopoda)
Bivalvia:	Only bivalves
Gastropoda:	Only gastropods
Bivalvia and Gastropoda:	At least one bivalve and one gastropod
Octopoda:	Both octopuses
Ecdysozoa:	Nematode and at least one insect
Insecta:	At least 2 insects
Deuterostomia:	At least one Ambulacraria and one Chordata
Chordata:	At least two chordates
Ambulacraria:	Both ambulacrarians

Block randomization

Random microsyntenic blocks were modelled after the distribution of observed microsyntenic blocks as described in [16]. Randomized syntenic blocks were computed as described in [16] (randomization for the phylogenetic tree was computed as described in [15] see below) Twenty randomizations were modelled after the distribution of either cephalopod-specific or metazoan synteny, resulting in 10101 and 5501 random blocks respectively.

3.2 Circos plot

Files were fomatted as follows: if a syntenic block was present in at least seven metazoans out of 24 species, it was counted as metazoan. If it was present in at least two cephalopods, but no other species, it was counted as cephalopod. Paralogous clusters were excluded. If a block was counted in at least five molluscs but no other species it was defined as mollusk specific. Results were then filtered to a subset of species to make the plot more easily readable. The circos plot was then plotted for 14 of the species in R using the circlize[17] package.

3.3 Density analysis of micro-syntenies, microsyteny distribution and karyoplots

The distribution of length in bp of metazoan microsyntenies and cephalopod-specific microsyntenies in *E. scolopes* (computed as described in 1.1-Microsyntenies) from start of first gene to start of last gene in the syntenic block was computed in with ggplot2's (https://ggplot2.tidyverse.org/) kernel density estimate. The same was done for the density of gene-counts of micro-syntenic blocks. Intergenic distances were calculated with a custom script, which uses a bed file as input that only contains whole genes (start-stop) and only the longest mapped transcripts for gene locations. Karyoplots for both *Mizuhopecten yassoensis* and *Euprymna scolopes* were plotted with KaryoplotR[18].

3.4 GO term enrichment analysis

Go term enrichment analysis of microsyntenic clusters was done with the topGO package[19]. *E. scolopes* protein sequences were annotated in Interpro[20] and all genes with GO term annotations were extracted. Cephalopod-specific and metazoan genes and their go annotations were compared to a list of all genes in the genome using the weight01 algorithm and fisher statistic parameters following the topGO manual.

Supplementary Note 4. Chromatin conformation analysis

4.1 TAD prediction

TAD prediction was done in TADbit[21] and in HiCExplorer[22,23]. First all matrices produced in HiC-Pro were converted to square matrices using pythons pivot.table function for each chromosome separately. TAD boundaries were then computed for each chromosome using TADbits default algorithm following the TADbit tutorial. These were used for the tree-analysis. For HiCPlotter we followed the HicExplorer tutorial. These were used for Figure 4b and Supplementary Figure 10 d. Principal component analysis to predict A/B compartments on Hi-C interaction matrices was done via HiCExplorer package following tutorial with hicPCA and hicCompartmentalization functions.

4.2 Synteny and TAD composition

Mizuhopecten yessoensis and *E. scolopes* pseudo-chromosomes were plotted with KaryoplotR as described in 1.3. Euprymna Hi-C maps, RNA-seq and Atac-seq tracks were plotted with HiCExplorer. First, HiC-Pro output files were converted to h5 format with the hicConvertFormat function, specifying the chromosome of interest (`hicConvertFormat --matrices`

`Sample1_40000_iced.matrix --outFileName Sample1_40000_Esc_group1 --inputFormat hicpro --outputFormat h5 --chromosome Lachesis_group1__63_contigs__length_187967259 --bedFileHicpro Sample1_40000_abs.bed --resolutions 40000`).

Mapped RNA-seq bam files (see also Supplementary Note 8) were converted to bigwig format using samtools[24] and deeptools[25] bamCoverage *e.g.*: 1. sorting with samtools sort, indexing with samtools index, bamCoverage with deeptools. Genes were plotted as exons, converting the *E. scolopes* gff to bed. TAD boundaries were plotted as predicted by HiCExplorer (`hicFindTads -m 40000.h5 -–outPrefix TADlachsesisgroup1_40000 –correctForMultipleTesting`). ATAC-seq peaks (see Supplementary Note 8) were plotted for one of each sample using the Genrich (https://github.com/jsh58/Genrich) narrowpeak file. All of these were added to an *ini* file and then plotted following hicexplorers tutorial.

4.3 TAD averaging

To understand the distribution of microsyntenies within TADs (as predicted by HiC explorer) we found the center of each microsynteny and its location in a TAD, which was then normalized to be able to compare all syntenies. The position of microsyntenic clusters in averaged tads were computed for a resolution of 100000bp/bin. The normalized locations were calculated by:

normalized center of synteny = (center of synteny - start of TAD) / TAD length

4.4 Motif finding at TAD boundaries

To identify possible motifs enriched at TAD boundaries, the locations of tadbit predicted TAD boundaries were extracted and converted to a bed file, converting the startbin to start with

TAD start = (bin start) * 100000

TAD stop = (bin stop + 1) * 100000

The regions were then checked for enriched motifs using homer[26] `findMotifsGenome.pl` with default `-size (200)` parameter against the non-masked genome.

4.5 Protein analysis

An smc1 orthogroup was identified from the orthofinder output. CTCF and smc3 proteins could not be identified in orthogroups, thus smc3 sequences were downloaded from NCBI. CTCF sequences were kindly provided by [27] and only one sequence for each species was used. *E. scolopes* orthologues were identified using BLASTP[28,29] (version 2.8.1+). Sequences were

aligned with mafft[30] (version 7.427) using default high speed settings. Approximate maximum-likelihood trees where then generated in FastTree[31,32] (version 2.1.11) and visualized with FigTree (version 1.4.3, https://github.com/rambaut/figtree/). The *E. scolopes* CTCF sequence was uploaded to InterproScan web tool (https://www.ebi.ac.uk/interpro/search/sequence/) which identified all 11 C2H2 Zinkinger domains. CTCF sequences of *E. scolopes*, *Lottia gigantea*, *Pecten maximus*, *Homo sapiens* and *Mus musculus* were realigned in mafft and visualized and annotated in Jalview[33] (version 2.11.1.0) to identify the regulatory domains described by [34].

Supplementary Note 5. Three dimensional modelling and SASA analysis

Three dimensional model generation

The 3D structures of individual chromosomes were constructed using a home-built C++ software. Each chromosome has a beads-on-a-string representation and starts with a randomized conformation. Then, the time evolution of chromosome conformation is governed by the Newton equation of motion, with forces (detailed below) implemented to characterize the chromosome structural integrity ($\vec{F}_i^{ten}$), volume exclusion between spatially overlapping genomic sites ($\vec{F}_i^{rep}$), drag by nucleoplasm ($-\gamma \vec{v}_i$), and genomically distant interactions suggested by Hi-C ($\vec{F}_i^{Hi-C}$). Pseudo-energy of 3D chromosome conformations, calculated as the sum of kinetic and potential energy in the system, is monitored throughout the simulation as an indicator of convergence.

The chromatin bead dynamics

The dynamics of a coarse-grained chromatin bead is governed by the following Newtonian equation of motion:

$$m\vec{a}_i = -\gamma \vec{v}_i + \vec{F}_i^{rep} + \vec{F}_i^{ten} + \vec{F}_i^{Hi-C}$$

where $\vec{a}_i$ and $\vec{v}_i$ are the instantaneous acceleration and velocity of the bead, respectively; m is the mass of the bead; γ is the drag coefficient; $\vec{F}_i^{ref}$, $\vec{F}_i^{ten}$, and $\vec{F}_i^{Hi-C}$ are forces implemented in the model to characterize the mutual volume exclusion between beads, the interaction between genomically consecutive beads, and the interaction between genomically distant beads with high Hi-C frequency. Computationally, Verlet integration is applied to calculate the trajectories of chromosome beads over time.

The volume exclusion force

The volume exclusion between any two spatially overlapping beads is assumed linearly elastic. The contribution of this force to a bead is described by the following equation:

$$\vec{F}_i^{rep} = \sum_{j \neq i}^{N} K^{rep} \left(d_{i,j} - d_{rep0} \right) u_{i,j}, if\ d_{i,j} < d_{rep0} \left(2 * radius_{bead} \right),$$

Where $K^{rep}$is the spring contant reflective the imcompressibility of genetic content within the beads in contact; $d_{i,j}$is the distance between the centre of two consecutively connected beads$i$ and j; d_{rep0} is the rest length of the linearly elastic spring;$u_{i,j}$ is a unit vector pointing from bead i to bead j.

The chromatin tension force

The interaction between two genomically consecutive beads is assumed linearly elastic. The contribution of this force to a bead is described by the following equation:

$$\vec{F}_i^{ten} = K^{ten} \left(d_{i,i-1} - c_2 \right) u_{i,i-1} + K^{ten} \left(d_{i,i+1} - c_2 \right) u_{i,i+1}$$

Where $K^{ten}$is the spring constant of the inter-bead 'chromatin' linker, $d_{i,i+1}$ is the distance between the centre of two consecutively connected beads and $i+1$; c_2 is the rest length of the linearly elastic spring; $u_{i,i+1}$ is a unit vector pointing from bead i to bead $i+1$.

The Hi-C restraint force

The interaction between genomically distant beads is also assumed linearly elastic. The contribution of this force to a bead is described by the following equation:

$$\vec{F}_i^{Hi-C} = \sum_{j \neq i}^{M} K^{HiC} \left(d_{i,j} - d_{hic0} \right) u_{i,j}, if\ p_{i,j} > p_{rep0} \left(thresholdHiCfreq. \right)$$

Where K^{Hi-C} is a constant reflective of the constraint strength implied by Hi-C and applies to any pairs of coarse-grained beads that have pair-wise Hi-C frequency greater than a threshold value, namely, $p_{i,j} > p_{rep0}$; d_{hic0} is the rest length of the linearly elastic spring; $u_{i,j}$is a unit vector pointing from bead i to bead j.

Data preparation and modelling

Normalized sparse matrix from Hi-C experiment was parse into separate single chromosome matrices, containing only intra-chromosomal contacts without any scaffold interactions. We removed all inter-chromosomal interactions due to multi-cell nature of Hi-C experiment. Furthermore, an interaction frequency (IF) cut-off was applied to further filter out desired contacts that were used as spatial constraints for modelling. Specifically, cut-off with IF value 5, 10 and 'mean' (mean of IF values for each single chromosome contact matrix) was tested for modelling purposes. The total number of cis contacts per chromosome together with number of filtered contacts used as constraints for reconstruction with different Hi-C thresholds are shown in Supplementary Table 4.

Structural measurements of mapped syntenic blocks (SASA, coverage, depth) can be affected by the number of constraints and thus compactness of chromatin model (Supplementary Figure 5 e-f). We keep the selection criteria for Hi-C threshold consistent among individual chromosomes to mitigate this impact.

All chromosomes were reconstructed with 3 replicates and each model was initialized with different conformation based on principles of self-avoiding random walk (SAWR). We monitored total energy of the system throughout the reconstruction to observe convergence of the system, which was then accompanied by RMSD analysis across all the time point structures towards the final structure (Supplementary Figure 5 methods a-b). We run the reconstruction algorithm for 10 000 timesteps and the final chromosome structure of each replicate run was then taken for further analysis. In order to validate correlation of our models with Hi-C interaction frequency (IF) map, we calculated cosine similarity between IF contacts, which were selected as restraints for 3D modelling, and Euclidean distance of corresponding genomic position in the model (Supplementary Figure 5 c). In addition, we calculated the proportion of satisfied/violated contacts (Supplementary Figure 5 d).

Supplementary Table 4. Hi-C constraints applied for single chromosome model reconstruction.

Chromosomal scaffold	Total number of contacts	Number of contacts			Percentage out of all contacts		
		IF = Mean	IF = 5	IF = 10	Mean (%)	5(%)	10(%)
1	437779	61766	28065	15331	14.11	6.41	3.5
2	390729	52413	24055	13093	13.41	6.16	3.35
3	386101	52819	24553	13951	13.68	6.36	3.61
4	359731	53525	28355	14741	14.88	7.88	4.1
5	341396	49758	25359	13348	14.57	7.43	3.91
6	305013	45807	21228	10514	15.02	6.96	3.45
7	285481	38272	19353	10448	13.41	6.78	3.66
8	263006	37901	21140	11218	14.41	8.04	4.27
9	272957	40552	24197	12578	14.86	8.86	4.61
10	244832	35336	20964	10900	14.43	8.56	4.45
11	239624	35217	21644	11327	14.7	9.03	4.73
12	213560	31488	19380	9877	14.74	9.07	4.62
13	246373	36441	19606	10090	14.79	7.96	4.1
14	212316	29970	17775	9188	14.12	8.37	4.33
15	206384	30044	16761	8235	14.56	8.12	3.99
16	194760	27969	20081	10841	14.36	10.31	5.57
17	177195	26210	18246	8969	14.79	10.3	5.06
18	176919	26123	16475	7914	14.77	9.31	4.47
19	144672	20625	14515	7283	14.26	10.03	5.03
20	143840	21947	16694	8105	15.26	11.61	5.63
21	133516	19273	14970	6925	14.43	11.21	5.19
22	145243	22221	15662	7243	15.3	10.78	4.99
23	152636	22318	14310	7061	14.62	9.38	4.63
24	138917	22288	15437	6742	16.04	11.11	4.85
25	130575	20706	16447	7652	15.86	12.6	5.86
26	123211	18354	13246	5572	14.9	10.75	4.52
27	113320	19027	17453	8506	16.79	15.4	7.51
28	121773	17615	13997	7266	14.47	11.49	5.97
29	108909	14466	11355	5916	13.28	10.43	5.43
30	110198	16978	12731	5319	15.41	11.55	4.83
31	94703	14756	14383	6746	15.58	15.19	7.12

32	95536	15111	12277	5405	15.82	12.85	5.66
33	87705	13573	11546	5114	15.48	13.16	5.83
34	76196	12938	13860	6767	16.98	18.19	8.88
35	80592	12044	11309	5312	14.94	14.03	6.59
36	68242	8767	7794	3712	12.85	11.42	5.44
37	77952	11327	10687	5176	14.53	13.71	6.64
38	66148	10191	10393	4920	15.41	15.71	7.44
39	53203	8195	9258	3897	15.4	17.4	7.32
40	36828	5875	8203	3354	15.95	22.27	9.11
41	44046	7012	7948	2981	15.92	18.04	6.77
42	41701	7053	9055	3889	16.91	21.71	9.33
43	31861	4710	7575	3146	14.78	23.78	9.87
44	9209	1223	2670	1042	13.28	28.99	11.32

Model analysis

- *Surface accessible solvent area*

We modified the `freesasa` python package (https://freesasa.github.io/python/) in order to make it applicable for SASA calculations of 3D chromosome scaffold models. We also calculated 'normalized SASA', $SASA_n$, contribution by cephalopod or metazoan synteny per chromosome as:

$$SASA_n = \frac{SASA_i}{n}$$

where $SASA_i$ is SASA contribution of all metazoan or cephalopod microsynteny on selected chromosome scaffold, and *n* is the genomic size of cluster (bp).

In addition to normalization, we can calculate proportion coverage of chromosome scaffold surface $p_{coverage}$ as:

$$p_{coverage} = \frac{\sum SASA_{ceph_i}}{SASA_{chromosome}}; \; p_{coverage} = \frac{\sum SASA_{meta_i}}{SASA_{chromosome}}$$

where $SASA_{chromosome}$ is the total SASA of chromosome and $\sum SASA_{ceph_i}$ (or $\sum SASA_{meta_i}$) is the sum of all the SASA of cephalopod/metazoan clusters on the particular chromosome scaffold.

Alternatively, we can calculate the proportional coverage $p_{coverage_i}$ of chromosome scaffold surface by occupied each individual microsynteny cluster:

$$p_{coverage_i} = \frac{SASA_{ceph_i}}{SASA_{chromosome}}; \quad p_{coverage_i} = \frac{SASA_{meta_i}}{SASA_{chromosome}}$$

- *Cluster depth*

Depth of cluster is defined as the distance between centre of mass of the microsynteny cluster $d_{ceph} \vee d_{meta}$ and the closest point of chromosome scaffold located on its surface, .

$$D(d_{meta}, d_{surface}) = \sqrt{(d_{meta_1} - d_{surface_1})^2 + (d_{meta_2} - d_{surface_2})^2 + (d_{meta_3} - d_{surface_3})^2}$$
$$D(d_{ceph}, d_{surface}) = \sqrt{(d_{ceph_1} - d_{surface_1})^2 + (d_{ceph_2} - d_{surface_2})^2 + (d_{ceph_3} - d_{surface_3})^2}$$

Cluster depth is an exposure measure complementing the information provided by SASA.

Supplementary Note 6. Neighbour-joining method for TAD syntenic consistency profiling

All scripts for this part can be found in the folder `Tree_method` directory in the bitbucket repository. Only chromosome scale scaffolds were considered for this analysis. Unassigned and unordered scaffolds were filtered out prior to analysis. The normalized Hi-C matrix and bed files (HiC-Pro output) were split into separate matrix and bed files for each chromosome and sorted by the stop column. “Interaction trees” for each chromosome were computed. Using the intensity of interaction between bins we clustered pairs of bins together; two bins with the highest interaction become "sister groups" to each other, then they cluster with the bins with the next highest interaction until all bins are integrated in the tree. Every chromosome is saved as a newick tree file and the length of the branches indicate interaction intensities.

To understand how well a region is defined by its interactions we extracted the last common ancestor of that region (the bins in that region) from the whole tree for a chromosome. We then constructed a table with information about the syntenic clusters and their sub-tree structure *e.g.* synteny-type (cephalopod-specific, ancestral (metazoan) synteny), chromosome, synt-id,

farthest node, distance root farthest node, distance root to farthest leaf, number of nodes in the extr tree, number of nodes in the microsyntenic cluster, number of genes, bins total on that chromosome, count, number of leaves in the extracted tree (for 503 of the 505 cephalopod-specific clusters, 274 out of the 275 metazoan clusters, 10074 of random-cephalopod clusters and 5490 of random-metazoan clusters, as not all sub-trees could be extracted).

We calculated the ratio between the number of bins in the extracted tree for a syntenic cluster (by last common ancestor) and the number of initial bins in the syntenic cluster. If a synteny location is well defined by its interaction, the ratio between the nodes of the tree and the bins making up the synteny id should be close to 1. Ratios higher than 1 were excluded. Wilcox test (unpaired, two-sided) was used to test differences between samples, using ggstatsplot (https://github.com/IndrajeetPatil/ggstatsplot). Different parameters were tested: using the whole matrix, filtering for clusters with at least 4 genes, using only clusters within a certain size range: minbin 15 and max bin 50 for 20 KBp resolution, minbin 7 and maxbin 25 for 40 KBp resolution and minbin 3 and maxbin 10 for 100 KBp resolution - this seems to be the best solution as it reduces the bias that the random syntenies are generally larger than the observed syntenies and was used in the presented plots for 40 KBp matrices. Results with 1 MBp and 20 KBp resolution were less robust. Heatmaps with trees were plotted in R for a resolution of 100 KBp.

Supplementary Note 7. Co-expression analysis and GO term enrichment

To test if the expression of genes in microsyntenic clusters is enriched in specific tissues we annotated genes to either cephalopod-specific or metazoan microsynteny and extracted the TMM expression values of adult *E. scolopes* tissues[9]. Genes with zero expression were excluded. We then calculated the tau value[35] for each of the annotated genes. Genes with a tau value of >=0.8 were extracted. The proportion to which each of the tissues contributes to the overall expression was calculated and for each gene, the tissue with the highest contribution was counted. No clear enrichment for one of the microsyntenic types in a nervous tissue could be found.

To further understand the contribution of microsyntenic expression to different tissues we calculated the mean expression of all genes in a microsyntenic cluster. Genes with zero expression were excluded and clusters with less than three genes after this filtering were also excluded. Additionally, hemocytes were removed from this analysis because hemocyte expression is generally very low. Mean expression of microsyntenic clusters was then z_scaled and plotted in R with the complex heatmap[36] function. Expression clusters were defined by splitting the dendrogram into 8 groups forming clear expression modules.

To test whether genes in microsyntenic clusters are more likely to be co-expressed than genes that randomly sit in close proximity in the genome we sampled the genome 20x by the distribution of cephalopod-specific and metazoan syntenies (number of genes)(see 2.1.), extracting clusters of genes in close proximity that are not syntenic. We then calculated the co-expression coefficient of genes in observed and random microsyntenies followin[16,] with updated the fisher correction. Again, hemocytes were excluded from the analysis and genes with zero expression were excluded as well as microsyntenic clusters with less than three genes after this filtering. Significance of results were tested with unpaired two-sided wilcox test in ggstatsplot. To compare the results with *Octopus,* orthologous microsyntenic clusters shared between *O. bimaculoides* and *E. scolopes* were extracted resulting in 471 cephalopod-specific microsyntenic clusters in *O. bimaculoides*. All microsyntenies shared between *O. bimaculoides* and 6 more species were used as metazoan clusters in *O. bimaculoides* to get a good sample size (293 clusters). Expression of the genes was then annotated and analysed as before. For the heatmap, only syntenies with a direct orthology between *E. scolopes* and *O. bimaculoides* were used and octopus syntenies were labelled with *E. scolopes* synteny identifier. This resulted in 448 cephalopod-specific and 143 metazoan syntenies in *O.bimaculoides* that were used to produce the expression heatmap.

Supplementary Note 8. Annotation of putative CNEs

The *E. scolopes* and *O. bimaculoides* and the *E. scolope*s and *A. dux*[37] genomes were aligned using *E. scolopes* as the query sequence using megablast. Five different settings for BLAST similarity scores (-perc_identity) were used: 0% , 70%, 80%, 95% and 98% (Supplementary table 5, see [38–41]). Other settings were: `-max_target_seqs 10 -max_hsps 1000 -task megablast -template_length 16 -penalty -2 -word_size 11 -evalue 1 -template_type coding_and_optimal.` Multimapping regions were excluded if they overlapped by more than 50% and occured more than 3 times using BEDOPS[42] `bedmap --count --echo --fraction-both 0.5 --delim '\t' prefiltered_megablast.bed | awk '$1<4' | cut -f2- |sort-bed - | uniq` and `bedops –merge` [35]. Any region overlapping with an exon by 1bp or more was excluded using bedtools[43] subtract with the -A parameter. To exclude repetitive regions, fasta sequences were extracted from the filtered putative CNE locations and meme's dust (cut-off 10) function was used to mask repeats. Any region with more than 25% Ns was excluded. Additionally, two datasets were created for each similarity score of at least 100bp or 50bp regions or if regions had fewer than 50 or 100bp non N nucleotides. For similarity scores of 0%, only 100bp regions

were kept (custom python script available on https://bitbucket.org/hannahschm/ceph_regulation_microsynteny/). To remove any remaining coding sequences, the remaining putative CNE sequences were blasted against the NCBI[44] NR database (mirrored on Mar 17 2021 for 0% similarity and Jan 3 2022 for others) and any regions overlapping with a BLAST match were removed with bedtools intersect -A.

Supplementary Table 5. CNE counts using different parameters for minimum length and similarity.

Similarity	Min size	Species	Number of CNEs	Overlap with atac-peaks
0	100bp	esc adu	46804	59
0,7	100bp	esc adu	42920	14
0,7	50bp	esc adu	93935	77
0,8	100bp	esc adu	42920	14
0,8	50bp	esc adu	93932	77
0,95	100bp	esc adu	42920	14
0,95	50bp	esc adu	93931	77
0,98	100bp	esc adu	42920	14
0,98	50bp	esc adu	93933	77
0	100bp	esc obi	2168	21
0,7	100bp	esc obi	1004	6
0,7	50bp	esc obi	10962	45
0,8	100bp	esc obi	921	9
0,8	50bp	esc obi	9172	48
0,95	100bp	esc obi	1187	12
0,95	50bp	esc obi	11605	48
0,98	100bp	esc obi	906	10
0,98	50bp	esc obi	9223	43

Supplementary Note 9. Chromatin accessibility assay by ATAC-seq

9.1 ATAC-seq library preparation

ATAC-seq library preparation was based on the OMNI-ATAC[45–47] seq method with modifications as described below. After removing the chorion and yolk sac, the embryos from stage 20, 24/25 and 28/29[40] were dissociated by Liberase treatment in PBS (1:100 dilution Unit of Liberase™, for 15 min at RT). Each library was obtained from 3 animals and 1.5 to 2 x10^4 K cells were used. After cell dissociation, the dissociated squid cell suspensions were spun down (380 x g for 5 min at 4°C) and re-suspend in 750 µl of ice-cold lysis buffer no.1 by gentle pipetting. Then cells were again spun down (380 x g for 5 min at 4°C) and pellets were resuspended in 100 µl of ice-cold lysis lysis buffer no.2 and incubated on ice 5 min. Ice cold lysis buffer no. 3 (750 µl) was added on top of cell mixture and mixed gently. Immediately, cells were spun down (380 x g for 5 min at 4°C). The cell pellets were resuspended with 50 µl of Tn5 Transposase reaction mixture (Transposase reaction: 5 µl of the 5x Transposase buffer (in-home): 50 mM TAPS-NaOH (pH 8.5), 25 mM $MgCl_2$, 50% DMF, 16.5 µl of PBS, 1 µl of 10% Tween-20 (0.1 % f. c.), 1 µl of 1% Digitonin (0.01 % f. c.), 5 µl of assembled in-home Tn5 (0.5 µg of assembled Tn5 was used for a reaction). Tn5 assembly with adapters was carried out following[6]. Transposition with Tn5 was carried out at 37°C for 1 hr with occasional agitation. The reaction was stopped by adding 5X the volume of PB buffer (QIAGEN) and vortexing for 30 second. Tn5 treated DNA was purified (QIAGEN mini purification kit) and eluted into 20 µl of EB buffer. Purified DNA solution (10 µl) was used for library amplification, and final amplification cycles were defined with intermediate qPCR[45,47.]. Final libraries were amplified with NEBNext® High-Fidelity 2X PCR Master Mix (NEB)). After PCR amplification, the libraries were purified with DNA purification beads. Sequencing was performed by 125 bp paired-end mode at VBCF NGS.

9.2 ATAC-seq - Mapping, quality control and peak calling

Quality of reads was assessed with fastqc (https://bioinformatics.babraham.ac.uk/projects/fastqc/). Trimming of reads was done with bbduk (`ktrim=r qtrim=15 k=21 mink=8 hdist=0`) from the bbtools package (https://jgi.doe.gov/data-and-tools/bbtools/bb-tools-user-guide/bbduk-guide/ version 38.56). Reads were mapped to the *E. scolopes* chromosomal assembly using bowtie2[11] (Version 2.3.5.1) (`--very-sensitive -k 10 -p 8`). Samtools[24] (Version 1.10) fixmate was run on aligned bam files, then the command “`samtools view -f $(samtools flags PROPER_PAIR,READ1 | cut -f 1) *.fixed.bam | awk '{print $9}'>`

`*insert_sizes.txt`" to extract insert sizes. Insert sizes were plotted in R. To get a better understanding of the location of peaks, we annotated the genome using PASA[48] (Version 2.3.3). The same transcripts that were used for the initial *E. scolopes* annotation were used and it was run on the new chromosomal assembly. PASA was run with `Launch_PASA_pipeline.pl -C -c PASAconfig.txt -R -g Euprymna_chormosomal_assembly.fasta -t Eup.final.fna --ALIGNER S gmap --CPU 8`. Afterwards, the output was compared to the published *E. scolopes* annotation and annotated with the same gene names. Then only the longest transcript for each gene was retained if there were still several isoforms left. The PASA annotation retained 16097 annotated genes (compared to 24378 in our original GFF file).

Peaks were called on aligned bam files with Genrich (`-j -y -r -v`) (https://github.com/jsh58/Genrich). Reproducible peaks were called from replicates using IDR (Irreproducible discovery rate, https://github.com/nboley/idr), resulting in 50-61.8% of peaks passing the IDR cutoff of 0.05. Peaks were annotated to genomic regions with ChIPseeker[49] using the new PASA annotation. Promoter regions were annotated as +10 kb and -10 kb from the transcription start site. If peaks were annotated to a gene belonging to a specific microsyntenic cluster, they were annotated to ceph/meta or non syntenic. Overlaps of peaks were plotted with limma[43], peaks overlapping between replicates were identified with soGGi's[50] `runConsensusRegions` function following the ATACseq in Bioconductor tutorial (https://rockefelleruniversity.github.io). All peaks overlapping with microsyntenic regions were extracted using the `findOverlaps` function of the GenomicRanges[51] package.

9.3 Motif finding ATAC-seq and repeat annotation of ATAC-peaks

To find any enriched motifs in regions of ATAC-seq peaks, locations of microsyntenic clusters falling into specific expression modules were extracted. Only peaks that were present in both replicates of ATAC-seq samples were used (but not IDR peaks). Those peaks were extracted using the bedtools[43] with the command `bedtools intersect -wa stage*_1.genrich_peaks -b stage*_2.genrich_peaks | sort | uniq > intersect_stage*.bed`, where `stage*_*` represent the respective replicates for each stage. Then, using bedtools intersect, regions of all microsyntenies falling into an expression module were compared to atac-seq peaks and intersecting regions were extracted e.g.: `bedtools intersect -a expression_module_*.bed -b intersect_stage*.bed> module_intersect_atac_stage*.bed`, where `expressionmodule*.bed` represents a bed file with the locations for all microsyntenic clusters falling into one of the expression modules.

Motifs were then identified for all ATAC peaks overlapping a specific cluster using homer`s `findMotifsGenome.pl` using the default size parameter (`=200`). Summarized results for p-values <=1e-3 are found in Supplementary Table 1. Motifs were annotated with information from the uniprot[50] website.

Transposable elements were annotated using the repeat element library of[9] with RepeatMasker (repeatmasker.org). Peaks overlapping syntenic clusters were extracted using bedtools intersect (`-F 1`). Total counts of individual repeat elements in ATAC and other regions were done with a custom script taking only one repeat annotation per base pair (to avoid overlapping annotations).

Supplementary Note 10. Orthologous expression (*Mizuhopecten yassoensis*) and co-expression analysis

RNA-seq from *Mizuhopecten yassoensis* was downloaded from NCBI (Supplementary Table 6).

Supplementary Table 6. *Mizuhopecten yessoensis* expression data used in this study

**Sequence Read Archive Number**	**Tissue**	**Trimming parameters**
SRR1185962	mantle from right shell (mantle_1)	`ktrim=r k=21 mink=11`
SRR441581	eye replicate 1	`ktrim=r qtrim=r trimq=15 k=21 mink=11 hdist=1`
SRR4428736	eye replicate 2	`ktrim=r trimq=10 k=21 mink=11 hdist=1`
SRR3289263	testis	`ktrim=r k=21 mink=11`
SRR4415816	gill replicate 3	`ktrim=r k=21 mink=11`
SRR4428737	mantle replicate 2	`ktrim=r k=21 mink=11`
SRR4428738	gill replicate 2	`ktrim=r k=21 mink=11`
SRR6407589	nerve ganglia	`ktrim=r k=21 mink=11`
SRR7287159	hemocytes1	`ktrim=r k=21 mink=11`
SRR7287161	hemocytes2	`ktrim=r k=21 mink=11`

Quality of reads was assessed with fastqc and sequences were trimmed for quality, adapters and overrepresented sequences with bbduk. Transcripts were quantified using Kallisto[52] for both *Mizuhopecten yassoensis* available on NCBI using the following commands:

```
kallisto index -i pye_rnaGCA.idx GCA_002113885.2_ASM211388v2_rna_from_genomic.fna
kallisto index -i pye_rnaGCF.idx GCF_002113885.1_ASM211388v2_rna.fna.gz
kallisto quant -i pye_rnaGCF.idx -o out.kallisto 1.fastq.gz 2.fastq.gz#
```

. For plotting, one of each replicates was used.

Orthologs between *E. scolopes* and *M. yassoensis* were identified from the orthofinder run (see section 2.1).

Supplementary Note 11. *In situ* hybridization and fluorescence *in situ hybridisation*

11.1 Probe synthesis

Different developmental stages of *Euprymna scolopes* were pooled, RNA was extracted with TRIzol as described in 6.2 and used to prepare cDNA using the SuperScript™ III CellsDirect™ cDNA Synthesis Kit (Invitrogen (Carlsbad, California, United States)). CDS and mRNA sequences from adult *E. scolopes* transcriptomes[9] were used to identify primer sequences. Primers were designed with Primer3[52,53] (gc clamp option) and inspected in primerstats[54].

Supplementary Table 7 Primer sequences and product sizes

Gene	Primer sequences (forward, reverse)	Product size
phenylalanine.tRNA ligase beta subunit-like (Gene_name: cluster_7042)	ACACCGATGATGAATTTGCCG<br>GGGTAAGAGAGTTGTCCTGGC	1034 bp
ceramide-1-phosphate transfer protein-like (Gene_name: cluster_9602)	CACCGAGTGAACCATCTCCC<br>TCTACGACGTGCATCAAG	1111 bp

	GC	
ceramide-1-phosphate transfer protein-like (Gene_name: cluster_14681)	TACCCGTATTTGCACTCGGC TCTTCATCACCAGAGACACGC	991 bp
amyloid protein-binding protein 2-like (Gene_name: cluster_9751)	ACTCGGAACTTCTCTTGCGG TCAAGTCCACTGTAACCGGC	1179 bp
splicing factor 3B subunit 4-like (Gene_name: cluster_10835)	TGAGGAAGATGCTGACTATGCG CTCGTTGTTGAGGTGGAGGG	1031 bp
Beta-tubulin (control)	GTACAAGCTGGACAGTGTGG ATGTTCAGGCGAAAAGCTTTC	1164 bp

Sequences were amplified with Q5 high-fidelity DNA polymerase (NEB) according to the manufacturer's instructions. Annealing temperatures were determined with NEB™ calculator (http://tmcalculator.neb.com/). PCR products were gel eluted with a PeqGold elution kit (Peqlab (Erlaangen, Germany)). PCR products were ligated in pjet1.2 blunt cloning vectors (Thermo Fisher) using 0,5 µl 10x ligation buffer, 0.5 µl pjet vector, 0.5 µl T4 DNA ligase and 1-3,5 µl purified PCR product per gene, adding water to reach a total volume of 5 µl. Vectors were transformed into competent *E. coli* cells (TOP 10, Thermo Fisher) through heat shock. After adding 250 µl SOC medium, cells were incubated in a thermomixer for 30 min at 37°C and subsequently transferred to pre-warmed LB-AMP plates (100 mg/mL ampicillin) and incubated overnight at 37°C. Colonies were checked for inserts via colony PCR using standard pJet1.2 primers and Taq polymerase (NEB) and positive colonies with inserts of the right size were inoculated overnight. DNA was isolated with an innuPREP Plasmid Mini Kit (Analytik Jena (Jane, Germany)). Plasmid were Sanger sequenced at Microsynth (Vienna, Austria) to check insert direction and verify the sequences using Ape (https://jorgensen.biology.utah.edu/wayned/ape/ version 2.0). Plasmids were then diluted 1:1000, amplified with T7 and pJetR, or pJetF and Sp6 outer primer, depending on direction and

gel purified with the peqGold Gel Extraction Kit (Peqlab). Probes were synthesized with sp6 or t7 enzyme, depending on direction. 0.5µl DIG RNA labelling mix (NEB), 10x RNase Polymerase Reaction Buffer (NEB), enzyme (Sp6 or T7, NEB) and RNAse out (Roche (Basel, Switzerland)) were mixed with 3 µl DNA and incubated at 37°C overnight. 1 µl Turbo DNAse was added the next day and left to rest for 15-30 minutes. Afterwards 39 µl RNAse free water (double-distilled, Sigma-Aldrich), and 5 µl 8 M lithiumchloride were mixed in, followed immediately by 3x the volume ice cold EtOH 100%. The mix was spun at 4°C for 15 minutes at max. speed (14,000xg). Supernatant was removed, the pellet washed in 200 µl, spun again for 5 min and dried. Then the pellet was resuspended in 45 µl water and all steps except the washing step were repeated. The pellet was resuspended in 25 µl formamide and 25 µl water and run on a gel to estimate concentration and quality. Final probes were stored at -80°C until used.

11.2 *In-situ* hybridization (ISH) and fluorescence *in-situ* hybridization (FISH)

The following primary steps were the same for both FISH and ISH.

Negative controls were done for all genes using sense probes, as well as one round of adding all ingredients except probes. Probes were reused several times. In-situs were done on early (stage 20-23, middle (stage 24-26), late (stage 27-29)[55] embryos and hatchlings. Results for early and middle stages were very variable and are thus not included in the results. Hatchlings and some of the older embryos were treated with proteinase K (20 mg/ml stock diluted 1:2000 in PBS for 5-15min), washed in PBS 3x, postfixed in 4% PFA for 1h at RT, again washed 2x in PBS and transferred back to hybe for blocking.

Embryos were blocked in 2 ml pre-heated hybe buffer at 72°C for at least 1h or overnight. Then 10-20 µl probe was added and embryos were incubated overnight if blocking was 1 h, or for 24 hours if blocking was done overnight at 72°C. The next morning, samples were washed reducing the salt concentration in a step-wise fashion. Washing was started with pre-heated hybe for 20 min, followed by 15 min washing steps of hybe and solution X (50% formamide, 2xSSC, 1% SDS, DEPC water)(50%:50%), hybe and solution X (25%:75%), solution X (100%), solution X and 0.05X SSC mix (0.05% SSC, 50% formamide, 50% DEPC water) (75%:25%), solution X and 0.05X SSC mix (50%:50%), solution X and 0.05X SSC mix(25%:75%), 0.05 ssc mix for 30 min (100%), all at 72°C. Then samples were washed at room temperature in 0.05 ssc and TBST (20 ml of 10x TBS (250 mM TrisHCl, pH 7.5, 1.36 M NaCl, 26.8 mM KCl, DEPC water), 200 ml of DEPC water; 2.5 ml of TWEEN® 20 (Sigma-Aldrich)) (75:25,50:50,25:75 for 10 min each, 100% TBST 4x for 5min each). Samples were blocked for 2h at RT in DIG 10-20% blocking solution (Roche) in TBST. In the meantime, powdered *E. scolopes* tissue in 500 µl

TBST was incubated at 72°C for 30 min, cooled on ice for 15 min and mixed with DIG blocking buffer (Roche). 10 µl DIG antibody (Sigma-Aldrich) was added to the solution and incubated for 1 h.

Steps for FISH

The antibody was then added to samples in blocking buffer (Roche) in a dilution of 1:500. Samples were incubated in Anti-Digoxigenin-POD (Sigma-Aldrich) antibody overnight at 4°C. Antibody incubation was followed by 1x quick, 3x 5min and 5x 1h washing steps in TBST. Samples were incubated in 200µl 1x Plus Amplification diluent (PerkinElmer (Waltham, Massachusetts, United Stages)) for 5min at RT, then 4 µl Cyanine 5 amplification reagent (Cy5) (PerkinElmer) was added and again embryos were incubated for 1-2h at RT on a rocker. Samples were washed 3x 15min in TBST, followed by two washing steps at 72°, first in detergent, then 30min in Solution X and 3x 15min in TBST at RT. Samples were stored in TBST at 4° until imaging. Nuclei were stained with DAPI (Sigma-Aldrich) and samples were mounted in Fluoromount-G® (SouthernBioTech (Birmingham, Alabama, United States)). Samples were imaged on an inverted Zeiss (Oberkochen, Germany) LSM780 multiphoton laser scanning confocal microscope at the Marine Biological Laboratory in Woods Hole.

Steps for ISH

The antibody was then added to samples in a blocking buffer (Roche) in a dilution of 1:5000. Samples were incubated in Anti-Digoxigenin AP-Conjugate (Sigma-Aldrich) overnight at 4C° followed by one quick, 3x 5min and 5x 1h washes in TBST. Samples were left overnight in TBST. Then samples were washed 3x in NTMT (100 mM NaCl, 100 mM Tris HCl (pH 9.5), 50 mM MgCl2, 1% Tween in DEPC water) and transferred to 3.5µL NBT (Thomas Scientific (Swedesboro, New Jersey, United States)) (100 mg/ml in 70% DMF/30% DEPC-H_2O) and 3.5µL BCIP (Thomas Scientific) (50 mg/ml in 100% DMF)/mL in NTMT. Color was left to develop for 2h-5 days and checked regularly. Samples were embedded in 10% gelatine solution in PBS. *E. scolopes* embryos were infiltrated for 1h at 37°C, then embedded in gelatine in custom molds. Gelatine molds were postfixed overnight in 3.7% PFA, washed 2x in PBS and sectioned (50µm) on a Leica VT 1200 S vibratome (Wetzlar, Germany). Sections were imaged on a Nikon Eclipse 80i microscope (Minato City, Tokyo, Japan).

Supplementary Table 8 TPM values of β-tubulin in late *E. scolopes* developmental stages

Stage27_1	Stage27_2	Stage27_3	Stage29_1	Stage29_2	Stage29_3
807.3973114	704.099415	694.6796078	743.794723	656.6387835	702.6270866

Supplementary Table 9 TMM values of β-tubulin in adult *E. scolopes*

Accessory nidamental gland	Brain	Eyes	Gills	Hemocytes	Light Organ	Skin
152.279	849.056	861.918	157.831	0.000	472.795	394.792

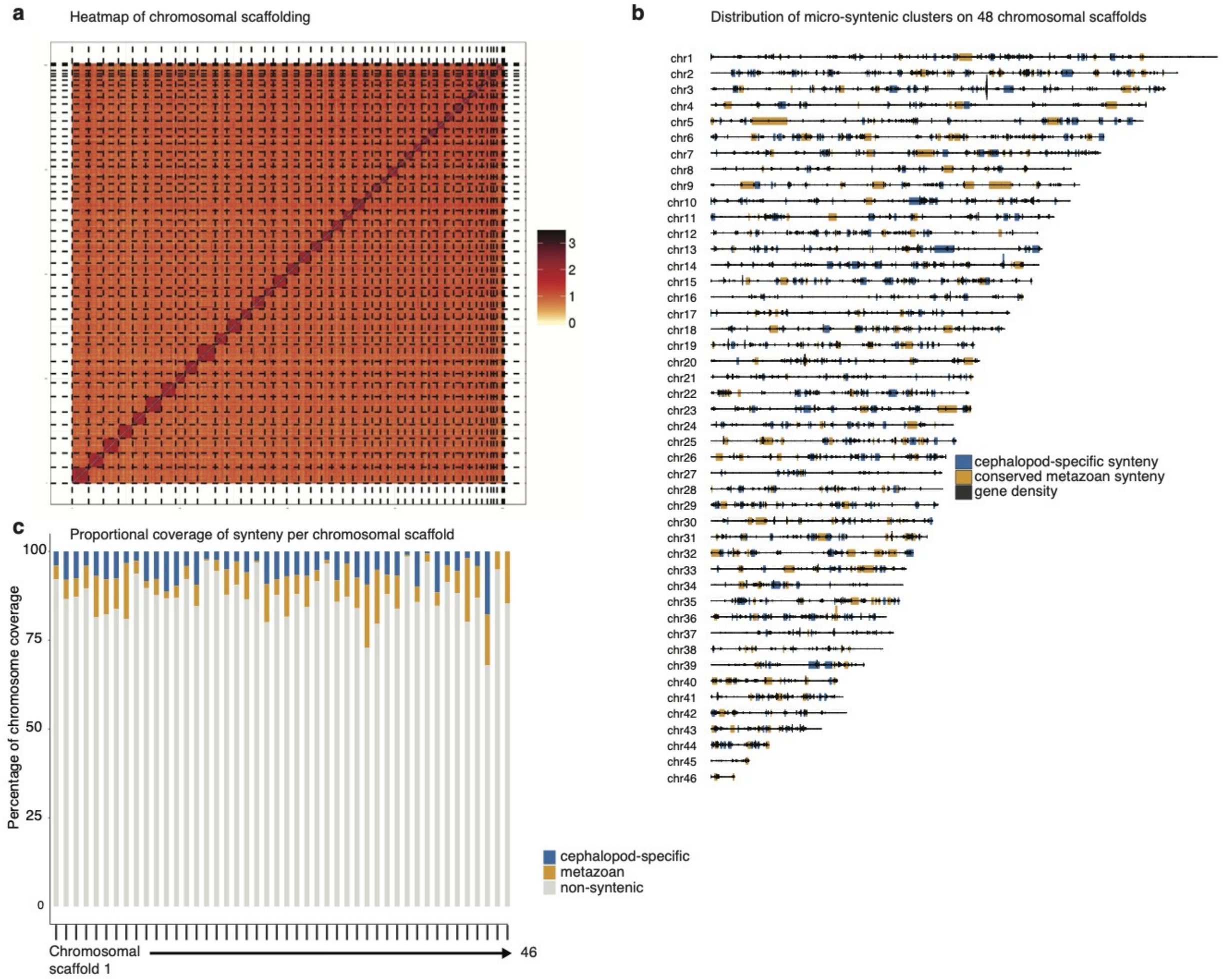

Supplementary Figure 1: Chromosomal-scale assembly of *Euprymna scolopes* identifying 46 chromosomal scaffolds. (a) Heatmap of Hi-C interaction densities of Lachesis clustering showing chromosomal scaffolds of *Euprymna scolopes*. (b) Distribution of cephalopod-specific (blue) and conserved, metazoan (orange) microsynteny on the 46 chromosomal scaffolds. Gene-density is plotted in black. (c) Proportion of coverage of microsyntenic clusters in bp per chromosome.

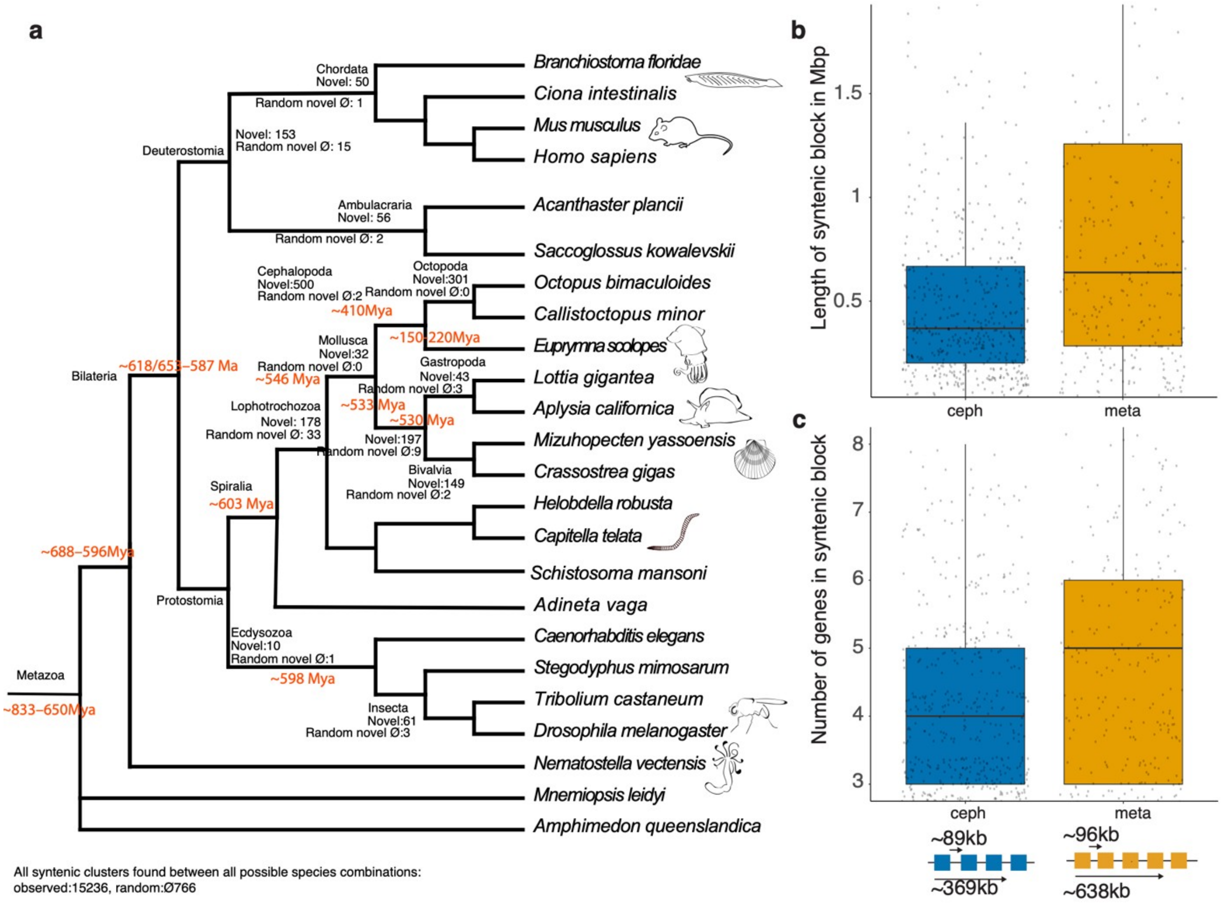

Supplementary Figure 2: Phylogenetic distribution and characteristics of microsyntenies. (a) Microsyntenic gain at the base of coleoid cephalopods. Total gains of microsynteny mapped onto the predefined tree of 24 species, representing all species used for synteny analysis. Divergence times after[55–57], relationships after [57]. (b) Boxplot of syntenic block sizes in cephalopod-specific syntenies present in *Euprymna scolopes* (blue, cephalopod-specific, n=505) and metazoan syntenies present in *E. scolopes* (yellow, metazoan, n=275, outliers not shown). Boxes - furthest sample within 1.5x interquartile range (cephalopod = min 0.01Mbp, max 1,36Mbp, metazoan = min 0.004Mbp, max 2.61Mbp), bars – median (cephalopod = 0.37Mbp, metazoan = 0.64 Mbp). Outliers were excluded from these numbers. Maximum and minimum values: cephalopod = min 0.01Mbp, max 7.44Mbp, metazoan = min 0.004 Mbp, max 14.14Mbp. (c) Boxplot of number of genes (outliers not shown), median number of genes, size in bp and intergenic distances in bp in metazoan (yellow) and cephalopod-specific (blue) syntenies in *E.* scolopes. Boxes - furthest sample within 1.5x interquartile range (cephalopod = min 3, max 8, metazoan = min 3, max 10), bars – median (cephalopod = 4, metazoan = 5). Outliers were excluded from these numbers, thus median differs slightly from real median. Maximum and minimum values: cephalopod = min 3, max 17, metazoan = min 3, max 39.

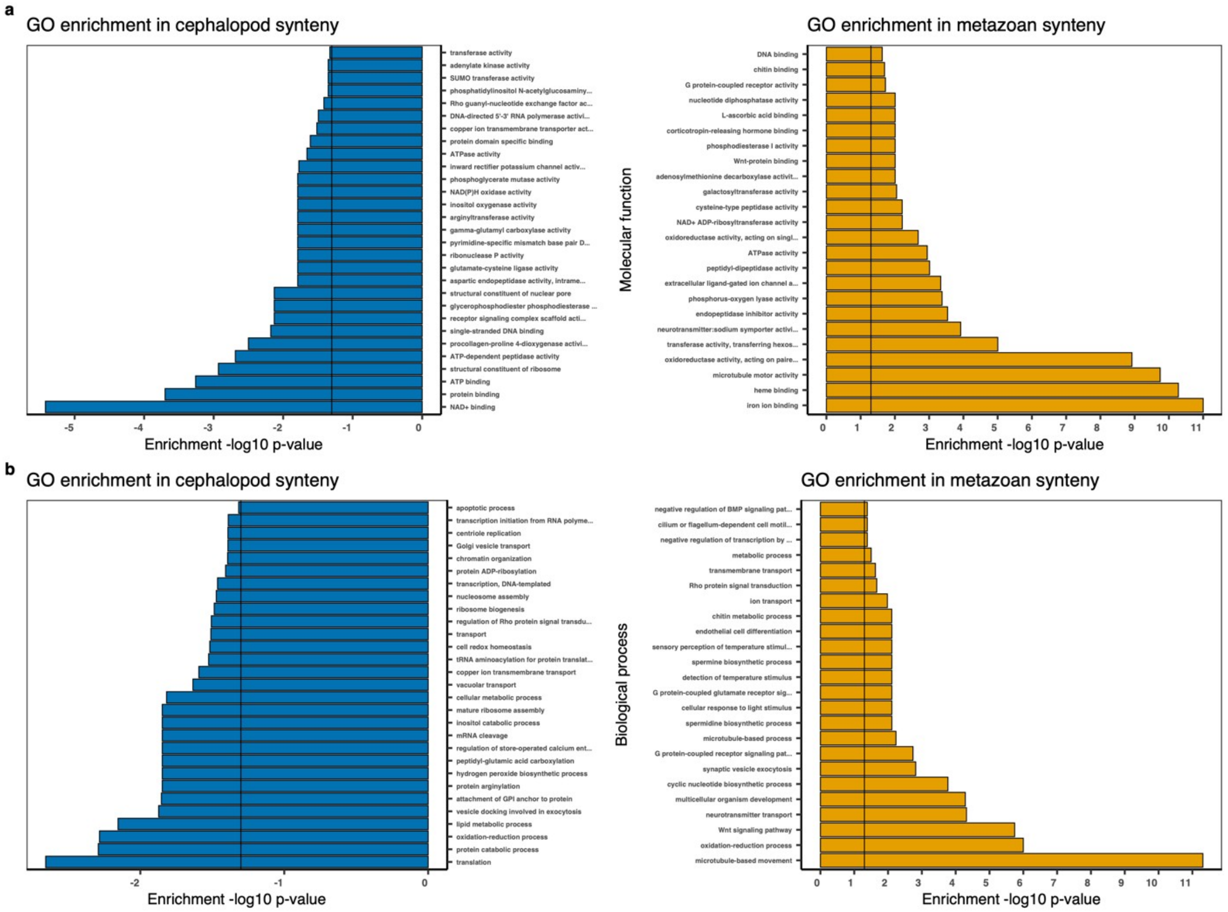

Supplementary Figure 3: Go-term enrichment analysis of microsyntenies in cephalopods. (a) Molecular function of enriched GO terms in cephalopod-specific (left) and metazoan (right) microsynteny. (b) Biological processes of enriched GO terms in cephalopod-specific (left) and metazoan (right) microsynteny. Black line: -log10 of p=0.05, y-axis: -log10 transformed p-values, x-axis: GO terms.

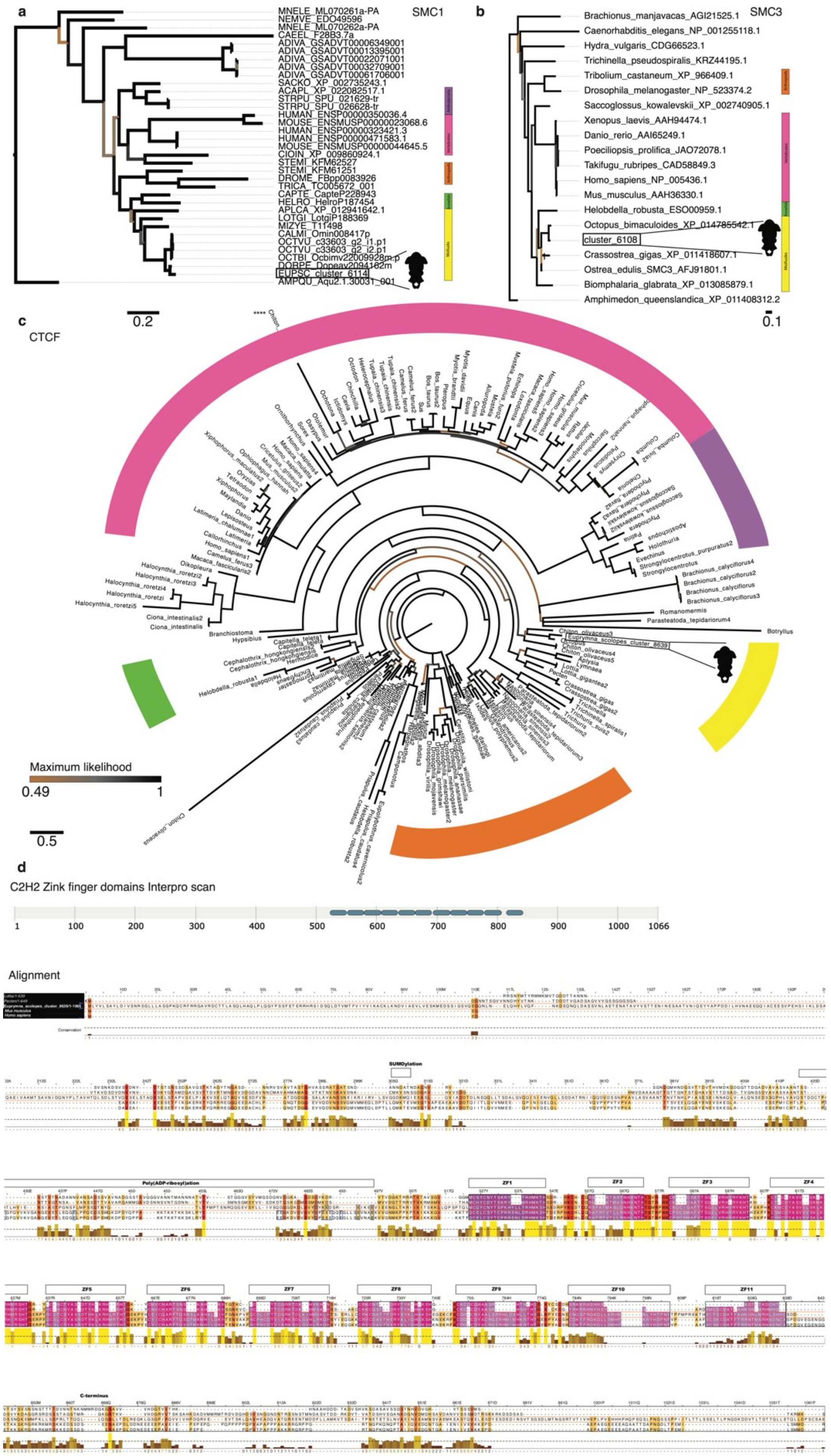
a
SMC1
MNELE_ML070261a-PA
NEMVE_EDO49596
MNELE_ML070262a-PA
CAEEL_F28B3.7a
ADIVA_GSADVT00006349001
ADIVA_GSADVT00013395001
ADIVA_GSADVT00022071001
ADIVA_GSADVT00032709001
ADIVA_GSADVT00061706001
SACKO_XP_002735243.1
ACAPL_XP_022082517.1
STRPU_SPU_021629-tr
STRPU_SPU_026628-tr
HUMAN_ENSP00000350036.4
MOUSE_ENSMUSP00000023068.6
HUMAN_ENSP00000323421.3
HUMAN_ENSP00000471583.1
MOUSE_ENSMUSP00000044645.5
CIOIN_XP_009860924.1
STEMI_KFM62527
STEMI_KFM61251
DROME_FBpp0083926
TRICA_TC005672_001
CAPTE_CapteP228943
HELRO_HelroP187454
APLCA_XP_012941642.1
LOTGI_LotgiP188369
MIZYE_T1I498
CALMI_Omin008417p
OCTVU_c33603_g2_i1.p1
OCTVU_c33603_g2_i2.p1
OCTBI_Ocbimv22009928m.p
DORPE_Dopeav2094182m
EUPSC_cluster_6114
AMPQU_Aqu2.1.30031_001
0.2
b
SMC3
Brachionus_manjavacas_AGI21525.1
Caenorhabditis_elegans_NP_001255118.1
Hydra_vulgaris_CDG66523.1
Trichinella_pseudospiralis_KRZ44195.1
Tribolium_castaneum_XP_966409.1
Drosophila_melanogaster_NP_523374.2
Saccoglossus_kowalevskii_XP_002740905.1
Xenopus_laevis_AAH94474.1
Danio_rerio_AAI65249.1
Poeciliopsis_prolifica_JAO72078.1
Takifugu_rubripes_CAD58849.3
Homo_sapiens_NP_005436.1
Mus_musculus_AAH36330.1
Helobdella_robusta_ESO00959.1
Octopus_bimaculoides_XP_014785542.1
cluster_6108
Crassostrea_gigas_XP_011418607.1
Ostrea_edulis_SMC3_AFJ91801.1
Biomphalaria_glabrata_XP_013085879.1
Amphimedon_queenslandica_XP_011408312.2
0.1
c
CTCF
Euprymna_scolopes_cluster_8639
Maximum likelihood
0.49
1
0.5
d
C2H2 Zink finger domains Interpro scan
1
100
200
300
400
500
600
700
800
900
1000
1066
Alignment
SUMOylation
Poly(ADP-ribosyl)ation
ZF1
ZF2
ZF3
ZF4
ZF5
ZF6
ZF7
ZF8
ZF9
ZF10
ZF11
C-terminus

Supplementary Figure 4: Smc1 and Smc3 cohesin subunits and CTCF complement in *Euprymna scolopes*. Maximum likelihood values are color-coded (gradient from 0.49-1).(a) Phylogenetic tree of SMC1 proteins, rooted by *A. queenslandica*. (b) Phylogenetic tree of SMC3 proteins, rooted by *A. queenslandica*. (c) Phylogenetic tree of CTCF proteins. The *E. scolopes* CTCF-sequence falls within CTCF of other mollusks. All 11 C2H2 zinc-finger domains were identified by Interpro. (d) Alignment of CTCF in Pecten (Scallop, Bivalvia), *Lottia* (Gastropoda), *E. scolopes*, Human and Mouse. C2H2 zinc finger domains are relatively conserved between species while other regions show high variability. 4 out of the 11 sites for poly(ADP ribosyl)ation (blue boxes) previously described to be essential for CTCF function in vertebrates,[47] are conserved in *E. scolopes*. Bars and colors indicate sequence conservation.

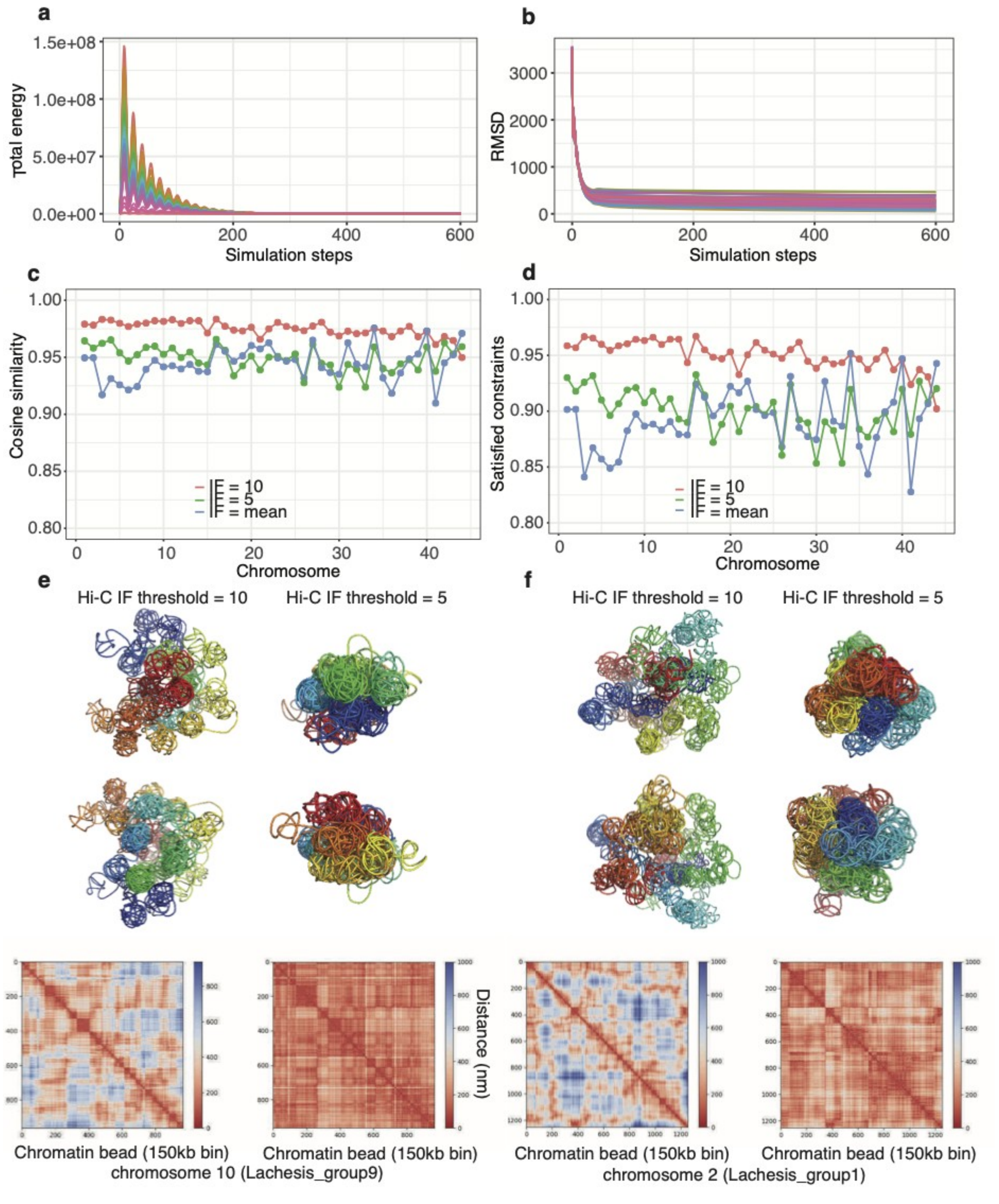

Supplementary Figure 5: Three dimensional modeling of *Euprymna scolopes* chromosomal scaffolds. (a) Convergence of total energy of the system for each chromosomal scaffold throughout the reconstruction. (b) RMSD convergence for each chromosomal scaffold throughout the reconstruction. (c) Cosine similarity between spatial distance constraints and selected Hi-C contacts for models reconstructed using different IF (Hi-C interaction frequency) cutoff (Supplementary Note 5). (d) Satisfaction of Hi-C derived constraints using different IF cutoff. (e) Three-dimensional model of chromosome 10 reconstructed with IF Hi-C cutoff 10

(left) and 5 (right), accompanied by distance heatmaps below. Higher number of Hi-C contacts results in three-dimensional models with much tighter packing. (f) Three-dimensional model of chromosome 2 reconstructed with IF Hi-C cutoff 10 (left) and 5 (right), accompanied by distance heatmaps below. Higher number of Hi-C contacts results in three-dimensional models with much tighter packing.

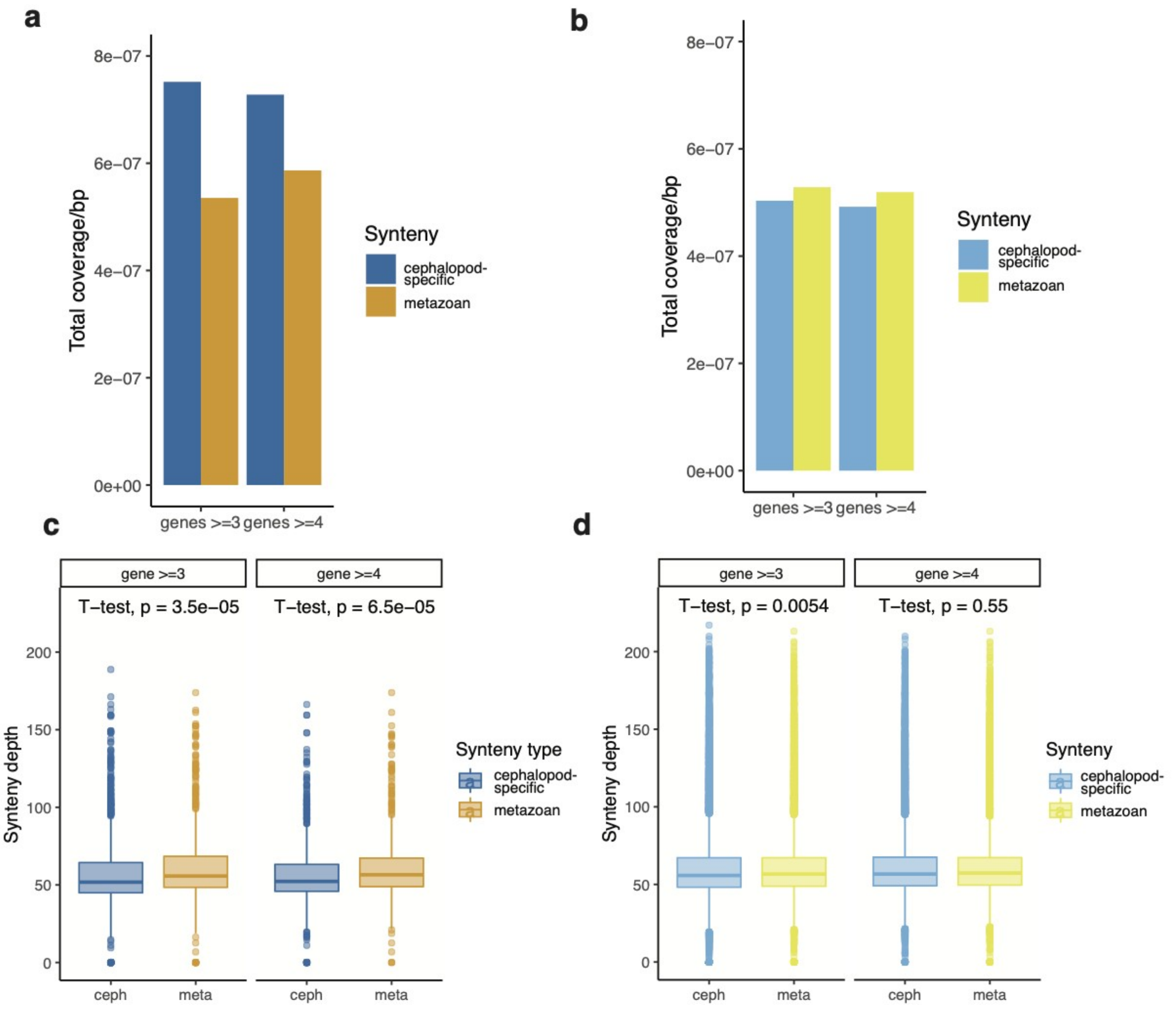

Supplementary Figure 6: Three dimensional properties of syntenies. (a) Total sum of SASA (Surface accessible solvent area, coverage) per bp genome wide for observed metazoan and cephalopod syntenies. (b) Total sum of SASA coverage per bp genome wide for randomized metazoan and cephalopod syntenies. (c) Distribution of observed metazoan and cephalopod-specific synteny depth. Metazoan synteny are localized significantly deeper within chromosomes. (d) Distribution of randomized metazoan and cephalopod-specific synteny depth. Metazoan and cephalopod-specific synteny consisting of at least three or four genes are shown and manifest similar trends. Boxes depict interquartile ranges and medians inside; whiskers reach out to lowest and highest values, respectively, with outliers represented as dots; all following the Tukey set of rules for boxplot representation (N_{ceph} = 1010; N_{meta} = 544 for synteny

with at least 3 genes and N_{ceph} = 1156; N_{meta} = 744 for synteny with at least 4 genes).

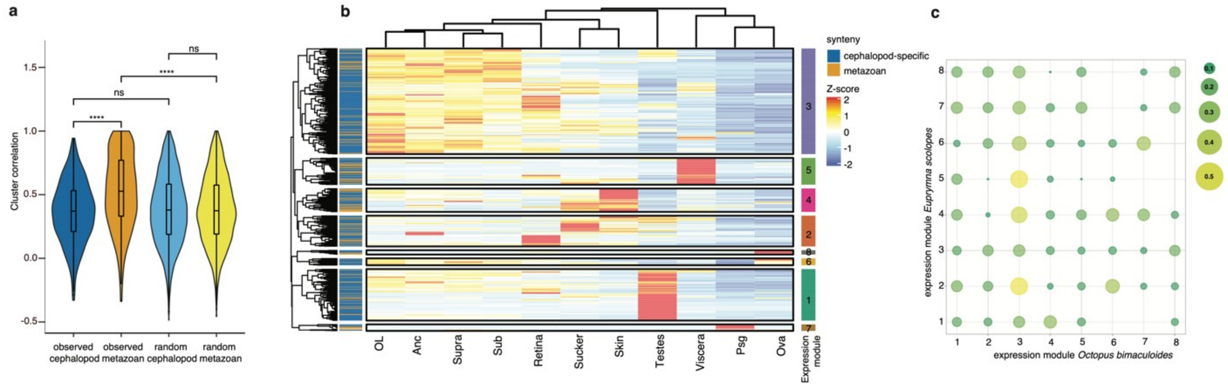

Supplementary Figure 7: Co-expression of syntenic genes and their expression modules in *Octopus bimaculoides*. (a) Cluster correlation of co-expression of genes in microsyntenic clusters in *O. bimaculoides*. The co-expression correlation of metazoan syntenies (n= 144) is significantly higher than that of cephalopod-specific syntenies (n= 169) or random clusters (random cephalopod n = 4368, random metazoan n = 1716), similar to results in *E. scolopes* (****p<0.0001). Boxes – furthest sample within 1.5x interquartile range, whiskers – cephalopod = min -0.26, max 0.94, metazoan = min 0.2, max 1.0, random cephalopod = min -0.4, max 1.0, random metazoan = min -0.37, max = 1.0), lines = median (cephalopod = 0.37, metazoan = 0.53, random cephalopod = 0.38, random metazoan = 0,37). Outliers were excluded from these numbers, thus median differs slightly from real median. Maximum and minimum correlation means: cephalopod = min -0.33, max 0.94, metazoan = min -0.34, max 1.0, random cephalopod = min 0.46, max 1.0, random metazoan = min 0.45, max 1.0. (b) Clustering of mean expression per synteny cluster, color-coded by synteny type, expression among *O. bimaculoides* adult tissues. Only syntenic clusters also present in *E. scolopes* were used for the clustering. Syntenic clusters form 8 expression modules with specific expression patterns. (c) Bubble plot of syntenies shared between expression modules in *E. scolopes* compared to *O. bimaculoides*. Size and color code indicate number of shared syntenies per expression module, normalized by the smaller cluster size.

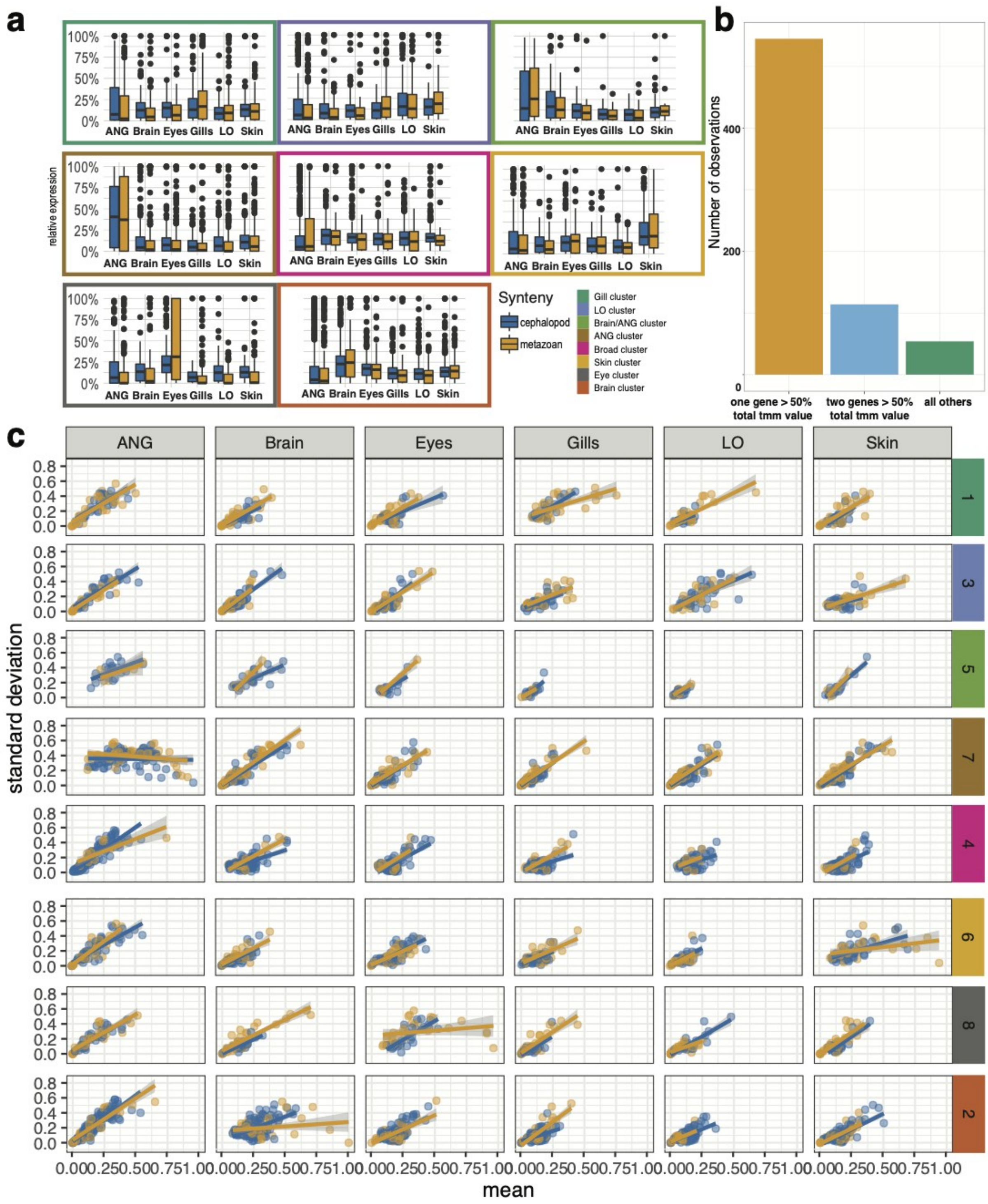

Supplementary Figure 8: Expression levels of genes within microsyntenies. (a) Distribution of normalized relative expression per tissue for each gene of microsyntenic clusters (genes in cephalopod synteny n= 12732, genes in metazoan synteny n = 7224) within each heatmap cluster of fig. 4d. Relative normalized expression correlates with mean expression values shown in the heatmap. Boxes - furthest sample within 1.5x interquartile range, lines = median. (b) Contribution of total expression level (tmm value) of genes in microsyntenic clusters. In most

microsyntenies, one gene contributes to more than 50% of the total tmm. (c) Standard deviation versus mean of syntenies labelled by clustering as shown in Fig. 4d. 95% confidence interval from linear model (grey).

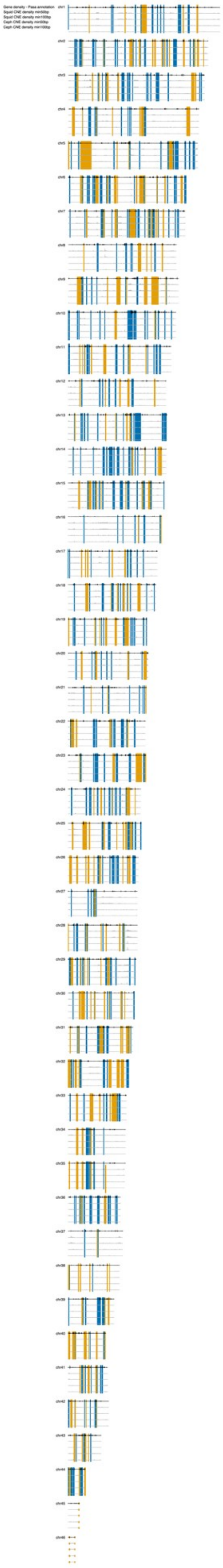

Gene density - Pasa annotation
Squid CNE density min50bp
Squid CNE density min100bp
Ceph CNE density min50bp
Ceph CNE density min100bp
chr1
chr2
chr3
chr4
chr5
chr6
chr7
chr8
chr9
chr10
chr11
chr12
chr13
chr14
chr15
chr16
chr17
chr18
chr19
chr20
chr21
chr22
chr23
chr24
chr25
chr26
chr27
chr28
chr29
chr30
chr31
chr32
chr33
chr34
chr35
chr36
chr37
chr38
chr39
chr40
chr41
chr42
chr43
chr44
chr45
chr46

Supplementary Figure 9: CNEs distribution along *Euprymna* chromosomes. Similarity threshold of 98% and different minimal lengths are plotted. Summary of similarity and length threshold effect on CNE counts is provided in Supplementary Table 5.

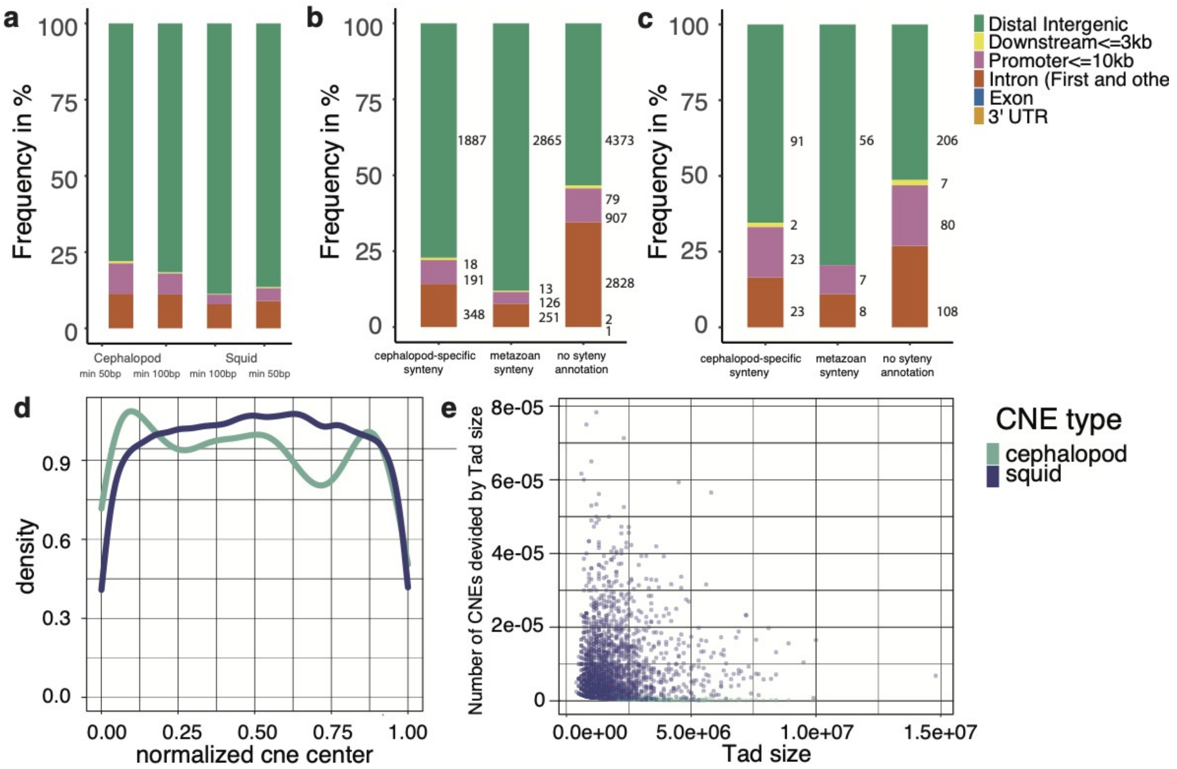

Supplementary Figure 10: CNEs in the *E. scolopes* genome. Cephalopod CNEs = shared between *E. scolopes* and *O. bimaculoides*. Squid CNEs = shared between *E. scolopes* and *A. dux* (a) Percentage of cephalopod and squid CNEs in different regions of the genome. (b) Relative frequencies of cephalopod CNEs to genes in different types of microsynteny (similarity 95%, min size 100bp). Plotted next to the bars are the absolute counts of regions that could be annotated. (c) Relative frequencies of squid CNEs to genes in different types of microsynteny (similarity 95%, min size 100bp). Plotted next to the bars are the absolute counts of regions that could be annotated. (d) Localization of CNEs in TADs. (e) Count of CNEs normalized by the TAD size they were located in.

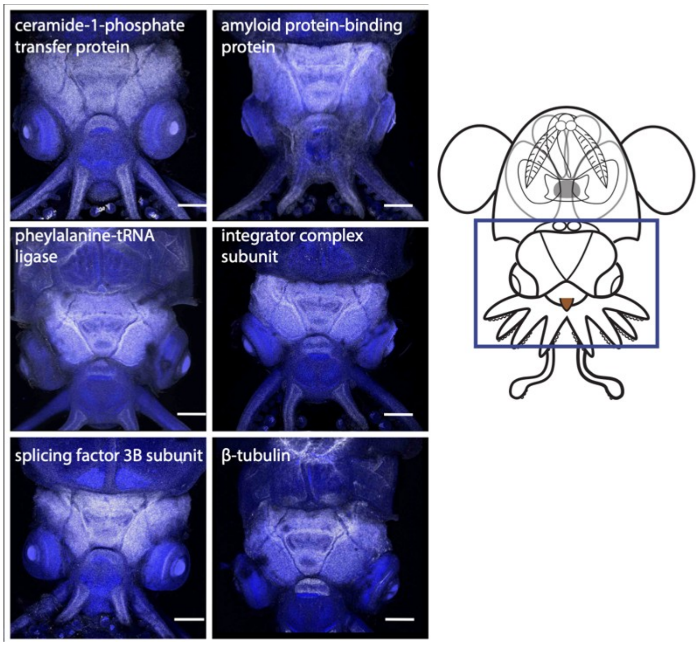

Supplementary Figure 11: Fluorescent in-situ hybridisation of the same genes as shown in Figure 5. Shown are only the head regions of late (Stage 27-29) embryonic stages. Scale bar=100µm

Supplementary references

1. Fiorito, G. *et al.* Guidelines for the Care and Welfare of Cephalopods in Research -A consensus based on an initiative by CephRes, FELASA and the Boyd Group. *Lab. Anim.* **49**, 1–90 (2015).
2. Butler-Struben, H. M., Brophy, S. M., Johnson, N. A. & Crook, R. J. In Vivo Recording of Neural and Behavioral Correlates of Anesthesia Induction, Reversal, and Euthanasia in Cephalopod Molluscs. *Front. Physiol.* **9**, (2018).
3. Shigeno, S., Andrews, P. L. R., Ponte, G. & Fiorito, G. Cephalopod Brains: An Overview of Current Knowledge to Facilitate Comparison With Vertebrates. *Front. Physiol.* **9**, (2018).
4. Collins, A. J. & Nyholm, S. V. Obtaining hemocytes from the Hawaiian bobtail squid *Euprymna scolopes* and observing their adherence to symbiotic and non-symbiotic bacteria. *J. Vis. Exp. JoVE* 1714 (2010) doi:10.3791/1714.
5. Wutz, G. *et al.* Topologically associating domains and chromatin loops depend on cohesin and are regulated by CTCF, WAPL, and PDS5 proteins. *EMBO J.* **36**, 3573–3599 (2017).
6. Picelli, S. *et al.* Tn5 transposase and tagmentation procedures for massively scaled sequencing projects. *Genome Res.* **24**, 2033–2040 (2014).
7. Nagano, T. *et al.* Comparison of Hi-C results using in-solution versus in-nucleus ligation. *Genome Biol.* **16**, 175 (2015).
8. Burton, J. N. *et al.* Chromosome-scale scaffolding of de novo genome assemblies based on chromatin interactions. *Nat. Biotechnol.* **31**, 1119–1125 (2013).
9. Belcaid, M. *et al.* Symbiotic organs shaped by distinct modes of genome evolution in cephalopods. *Proc. Natl. Acad. Sci.* **116**, 3030–3035 (2019).
10. Servant, N. *et al.* HiC-Pro: an optimized and flexible pipeline for Hi-C data processing. *Genome Biol.* **16**, 259 (2015).

11. Langmead, B. & Salzberg, S. L. Fast gapped-read alignment with Bowtie 2. *Nat. Methods* **9**, 357–359 (2012).

12. Emms, D. M. & Kelly, S. OrthoFinder: solving fundamental biases in whole genome comparisons dramatically improves orthogroup inference accuracy. *Genome Biol.* **16**, 157 (2015).

13. Emms, D. M. & Kelly, S. OrthoFinder: phylogenetic orthology inference for comparative genomics. *Genome Biol.* **20**, 238 (2019).

14. Camacho, C. *et al.* BLAST+: architecture and applications. *BMC Bioinformatics* **10**, 421 (2009).

15. Simakov, O. *et al.* Insights into bilaterian evolution from three spiralian genomes. *Nature* **493**, 526–531 (2013).

16. Zimmermann, B., Robert, N. S. M., Technau, U. & Simakov, O. Ancient animal genome architecture reflects cell type identities. *Nat. Ecol. Evol.* **3**, 1289–1293 (2019).

17. Gu, Z., Gu, L., Eils, R., Schlesner, M. & Brors, B. circlize implements and enhances circular visualization in R. *Bioinformatics* **30**, 2811–2812 (2014).

18. Gel, B. & Serra, E. karyoploteR: an R/Bioconductor package to plot customizable genomes displaying arbitrary data. *Bioinformatics* **33**, 3088–3090 (2017).

19. Alexa, A. & Rahnenfuhrer, J. topGO: Enrichment Analysis for Gene Ontology. R package version 2.40.0. (2020).

20. Mitchell, A. L. *et al.* InterPro in 2019: improving coverage, classification and access to protein sequence annotations. *Nucleic Acids Res.* **47**, D351–D360 (2019).

21. Serra, F. *et al.* Automatic analysis and 3D-modelling of Hi-C data using TADbit reveals structural features of the fly chromatin colors. *PLOS Comput. Biol.* **13**, e1005665 (2017).

22. Wolff, J. *et al.* Galaxy HiCExplorer: a web server for reproducible Hi-C data analysis, quality control and visualization. *Nucleic Acids Res.* **46**, W11–W16 (2018).

23. Wolff, J. *et al.* Galaxy HiCExplorer 3: a web server for reproducible Hi-C, capture Hi-C and single-cell Hi-C data analysis, quality control and visualization. *Nucleic Acids Res.* doi:10.1093/nar/gkaa220.

24. Li, H. *et al.* The Sequence Alignment/Map format and SAMtools. *Bioinformatics* **25**, 2078–2079 (2009).

25. Ramírez, F. *et al.* deepTools2: a next generation web server for deep-sequencing data analysis. *Nucleic Acids Res.* **44**, W160–W165 (2016).

26. Heinz, S. *et al.* Simple combinations of lineage-determining transcription factors prime cis-regulatory elements required for macrophage and B cell identities. *Mol. Cell* **38**, 576–589 (2010).

27. Heger, P., Zheng, W., Rottmann, A., Panfilio, K. A. & Wiehe, T. The genetic factors of bilaterian evolution. *Revis.* (2020).

28. Altschul, S. F. *et al.* Gapped BLAST and PSI-BLAST: a new generation of protein database search programs. *Nucleic Acids Res.* **25**, 3389–3402 (1997).

29. Schäffer, A. A. *et al.* Improving the accuracy of PSI-BLAST protein database searches with composition-based statistics and other refinements. *Nucleic Acids Res.* **29**, 2994–3005 (2001).

30. Katoh, K. & Standley, D. M. MAFFT Multiple Sequence Alignment Software Version 7: Improvements in Performance and Usability. *Mol. Biol. Evol.* **30**, 772–780 (2013).

31. Price, M. N., Dehal, P. S. & Arkin, A. P. FastTree 2--approximately maximum-likelihood trees for large alignments. *PloS One* **5**, e9490 (2010).

32. Price, M. N., Dehal, P. S. & Arkin, A. P. FastTree: computing large minimum evolution trees with profiles instead of a distance matrix. *Mol. Biol. Evol.* **26**, 1641–1650 (2009).

33. Waterhouse, A. M., Procter, J. B., Martin, D. M. A., Clamp, M. & Barton, G. J. Jalview Version 2--a multiple sequence alignment editor and analysis workbench. *Bioinforma. Oxf. Engl.* **25**, 1189–1191 (2009).

34. Pugacheva, E. M. *et al.* CTCF mediates chromatin looping via N-terminal domain-dependent cohesin retention. *Proc. Natl. Acad. Sci.* **117**, 2020–2031 (2020).

35. Kryuchkova-Mostacci, N. & Robinson-Rechavi, M. Tissue-Specificity of Gene Expression Diverges Slowly between Orthologs, and Rapidly between Paralogs. *PLOS Comput. Biol.* **12**, e1005274 (2016).

36. Gu, Z., Eils, R. & Schlesner, M. Complex heatmaps reveal patterns and correlations in multidimensional genomic data. *Bioinformatics* **32**, 2847–2849 (2016).

37. da Fonseca, R. R. *et al.* A draft genome sequence of the elusive giant squid, *Architeuthis dux*. *GigaScience* **9**, (2020).

38. Engström, P. G., Ho Sui, S. J., Drivenes, O., Becker, T. S. & Lenhard, B. Genomic regulatory blocks underlie extensive microsynteny conservation in insects. *Genome Res.* **17**, 1898–1908 (2007).

39. Kikuta, H. *et al.* Genomic regulatory blocks encompass multiple neighboring genes and maintain conserved synteny in vertebrates. *Genome Res.* **17**, 545–555 (2007).

40. Engström, P. G., Fredman, D. & Lenhard, B. Ancora: a web resource for exploring highly conserved noncoding elements and their association with developmental regulatory genes. *Genome Biol.* **9**, R34 (2008).

41. Bejerano, G. *et al.* Ultraconserved elements in the human genome. *Science* **304**, 1321–1325 (2004).

42. Neph, S. *et al.* BEDOPS: high-performance genomic feature operations. *Bioinformatics* **28**, 1919–1920 (2012).

43. Quinlan, A. R. & Hall, I. M. BEDTools: a flexible suite of utilities for comparing genomic features. *Bioinformatics* **26**, 841–842 (2010).

44. Sayers, E. W. *et al.* Database resources of the national center for biotechnology information. *Nucleic Acids Res.* **50**, D20–D26 (2022).

45. Buenrostro, J. D., Giresi, P. G., Zaba, L. C., Chang, H. Y. & Greenleaf, W. J. Transposition of native chromatin for fast and sensitive epigenomic profiling of open chromatin, DNA-binding proteins and nucleosome position. *Nat. Methods* **10**, 1213 (2013).

46. Corces, M. R. *et al.* An improved ATAC-seq protocol reduces background and enables interrogation of frozen tissues. *Nat. Methods* **14**, 959–962 (2017).

47. Buenrostro, J., Wu, B., Chang, H. & Greenleaf, W. ATAC-seq: A Method for Assaying Chromatin Accessibility Genome-Wide. *Curr. Protoc. Mol. Biol. Ed. Frederick M Ausubel Al* **109**, 21.29.1-21.29.9 (2015).

48. Dharmalingam, G. & Carroll, T. soGGi: Visualise ChIP-seq, MNase-seq and motif occurrence as aggregate plots Summarised Over Grouped Genomic Intervals, R package version 1.6.1. https://rdrr.io/bioc/soGGi/.

49. Lawrence, M. *et al.* Software for Computing and Annotating Genomic Ranges. *PLOS Comput. Biol.* **9**, e1003118 (2013).

50. Consortium, T. U. UniProt: a worldwide hub of protein knowledge. *Nucleic Acids Res.* **47**, D506 (2019).

51. Bray, N. L., Pimentel, H., Melsted, P. & Pachter, L. Near-optimal probabilistic RNA-seq quantification. *Nat. Biotechnol.* **34**, 525–527 (2016).

52. Koressaar, T. & Remm, M. Enhancements and modifications of primer design program Primer3. *Bioinformatics* **23**, 1289–1291 (2007).

53. Untergasser, A. *et al.* Primer3—new capabilities and interfaces. *Nucleic Acids Res.* **40**, e115–e115 (2012).

54. Stothard, P. The sequence manipulation suite: JavaScript programs for analyzing and formatting protein and DNA sequences. *BioTechniques* **28**, 1102, 1104 (2000).

55. Lee, P. N., Callaerts, P. & de Couet, H. G. The embryonic development of the Hawaiian bobtail squid (*Euprymna scolopes)*. *Cold Spring Harb. Protoc.* **2009**, pdb.ip77 (2009).
