# Supplementary material for: Emergence of novel cephalopod gene regulation and expression through large-scale genome reorganization
Source: Nat Commun. 2022 Apr 21;13:2172. doi: 10.1038/s41467-022-29694-7 (PMC9023564; doi:10.1038/s41467-022-29694-7)
Supplement: Supplementary file 3 — Description of additional Supplementary File [file 41467_2022_29694_MOESM3_ESM.pdf]

Descriptions of additional Supplementary data files

Supplementary Data 1: Detected microsyntenic blocks at cephalopod and metazoan nodes. PFAM annotation for Euprymna genes is provided in the last column.

Supplementary Data 2: Motif prediction and annotation in ATACSeq regions with $p<=0.001$
