# Supplementary material for: Emergence of novel cephalopod gene regulation and expression through large-scale genome reorganization
Source: Nat Commun. 2022 Apr 21;13:2172. doi: 10.1038/s41467-022-29694-7 (PMC9023564; doi:10.1038/s41467-022-29694-7)
Supplement: Supplementary file 6 — Reporting Summary [file 41467_2022_29694_MOESM6_ESM.pdf]

Corresponding author(s): Oleg Simakov
Last updated by author(s): 2020 07 30

Reporting Summary

Nature Research wishes to improve the reproducibility of the work that we publish. This form provides structure for consistency and transparency in reporting. For further information on Nature Research policies, see our Editorial Policies and the Editorial Policy Checklist.

Statistics

For all statistical analyses, confirm that the following items are present in the figure legend, table legend, main text, or Methods section.

n/a	Confirmed	
☐	☒	The exact sample size (*n*) for each experimental group/condition, given as a discrete number and unit of measurement
☐	☒	A statement on whether measurements were taken from distinct samples or whether the same sample was measured repeatedly
☐	☒	The statistical test(s) used AND whether they are one- or two-sided<br>*Only common tests should be described solely by name; describe more complex techniques in the Methods section.*
☐	☒	A description of all covariates tested
☐	☒	A description of any assumptions or corrections, such as tests of normality and adjustment for multiple comparisons
☐	☒	A full description of the statistical parameters including central tendency (e.g. means) or other basic estimates (e.g. regression coefficient) AND variation (e.g. standard deviation) or associated estimates of uncertainty (e.g. confidence intervals)
☐	☒	For null hypothesis testing, the test statistic (e.g. *F*, *t*, *r*) with confidence intervals, effect sizes, degrees of freedom and *P* value noted<br>*Give P values as exact values whenever suitable.*
☒	☐	For Bayesian analysis, information on the choice of priors and Markov chain Monte Carlo settings
☒	☐	For hierarchical and complex designs, identification of the appropriate level for tests and full reporting of outcomes
☒	☐	Estimates of effect sizes (e.g. Cohen's *d*, Pearson's *r*), indicating how they were calculated

Our web collection on statistics for biologists contains articles on many of the points above.

Software and code

Policy information about availability of computer code

Data collection

Described in Supplementary Note.

Data analysis

Bitbucket repository: bitbucket.org/hannahschm/ceph_regulation_microsynteny

Software and versions:
Python: PyCharm Community Edition 2017.2.3, Python 3.7.3, Python 2.7.16,
python3 packages/software: ete3 3.1.1
python2 ackages/software: tadbit 0.4.2

R: R studio Version 1.1.414, R Version 3.6.0
Libraries:
ggplot2 version 3.3.2
circlize package version 0.4.9.
gtools version 3.8.2
GenomicRanges version 1.36.1
karyoplotR version 1.10.5
DescTools version 0.99.35
tidyverse version 1.3.0
ggtree 1.16.6
tidytree 0.3.3
phylobase 0.8.10
maps 3.3.0

phytools 0.7.47
ape 5.4
gplots 3.0.3
tidyverse 1.3.0
dendextend 1.13.4
colormap 0.1.4
ggpubr 0.3.0
ggridges 0.5.2
argparse 2.0.1
fitdistrplus 1.1.1
cowplot 1.0.0

bedtools 2.27-2.29
samtools 1.12
hicexplorer 2.2.1.1
hicpro 2.11.1
lachesis (single version)
orthofinder 2.3.8

For manuscripts utilizing custom algorithms or software that are central to the research but not yet described in published literature, software must be made available to editors and reviewers. We strongly encourage code deposition in a community repository (e.g. GitHub). See the Nature Research guidelines for submitting code & software for further information.

Data

Policy information about availability of data

All manuscripts must include a data availability statement. This statement should provide the following information, where applicable:

- Accession codes, unique identifiers, or web links for publicly available datasets
- A list of figures that have associated raw data
- A description of any restrictions on data availability

The Hi-C and ATAC-seq data have been deposited in the NCBI database under Bioproject PRJNA661684. All expression, ATAC-seq, and CNE data that is mapped t the reference ge-nome is available on a genome browser (currently, http://metazoa.csb.univie.ac.at:8000/euprymna/jbrowse or upon request). Source data are provided with this paper. All other genomic and trascriptomic data used was downloaded from NCBI (GCA_002113885.2 (https://www.ncbi.nlm.nih.gov/assembly GCF_002113885.1/), GCA_000002075.2(https://www.ncbi.nlm.nih.gov/assembly/GCF_000002075.1/), GRCh38.p12 (https://www.ncbi.nlm.nih.gov/assembly/ GCF_000001405.38/), GCA_001949145.1 OLI-Apl_1.0 (https://www.ncbi.nlm.nih.gov/assembly/GCF_001949145.1/), GCA_000003605.1 (https:// www.ncbi.nlm.nih.gov/assembly/GCF_000003605.2), GCA_000224145.2 (https://www.ncbi.nlm.nih.gov/assembly/GCF_000224145.3), GCA_000003815.1 Versio (https://www.ncbi.nlm.nih.gov/assembly/GCF_000003815.1/#/st), GCA_004765925.1(https://www.ncbi.nlm.nih.gov/assembly/GCA_004765925.1/), Spur_3.1 (https://www.ncbi.nlm.nih.gov/assembly/GCF_000002235.3/), GRCm38.p6 (https://www.ncbi.nlm.nih.gov/assembly/GCF_000001635.26/), SAMN00691532 (https://www.ncbi.nlm.nih.gov/biosample/?term=SAMN00691532), SAMN00152410 (https://www.ncbi.nlm.nih.gov/biosample/SAMN00152410/)), ENSEMBL (BDGP6.28 (http://www.ensembl.org/Drosophila_melanogaster/Info/Index), WBcel235 (http://m.ensembl.org/Caenorhabditis_elegans/Info/Annotation), Capitella_teleta_v1.0 (http://metazoa.ensembl.org/Capitella_teleta/Info/Index), ASM23792v2 (http://metazoa.ensembl.org/Schistosoma_mansoni/Info/Index), oyster_v9 (http://metazoa.ensembl.org/Crassostrea_gigas/Info/Index), Helro1 (http://metazoa.ensembl.org/Helobdella_robusta/Info/Index), Lotgi1 (http:// metazoa.ensembl.org/Lottia_gigantea/Info/Index), PRJNA270931 (https://metazoa.ensembl.org/Octopus_bimaculoides/Info/Index), Stegody-phus_mimosarum_ (https://metazoa.ensembl.org/Stegodyphus_mimosarum/Info/Index), Tcas5.2 (http://metazoa.ensembl.org/Tribolium_castaneum/Info/Index), AMS_PRJEB1171_ (https://metazoa.ensembl.org/Adineta_vaga/Info/Index), GRCh37.p13 (https://grch37.ensembl.org/Homo_sapiens/Info/Index), ASM20922v1 (https:// metazoa.ensembl.org/Nematostella_vectensis/Info/Index), Aqu1 (https://metazoa.ensembl.org/Amphimedon_queenslandica/Info/Index), MnLei_Aug2011(http:// metazoa.ensembl.org/Mnemiopsis_leidyi/Info/Index)). Human Hi-C data was downloaded from NCBI (SRR1658570,HIC001(https://www.ncbi.nlm.nih.gov/sra/ SRR1658570)) or GIGA (PRJNA421033(http://gigadb.org/dataset/100503)). Processed files and tables needed to re-create the figures are accessible via a bitbuck repository:https://bitbucket.org/hannahschm/ceph_regulation_microsynteny/.

The bioproject PRJNA661684 has been made available.

Field-specific reporting

Please select the one below that is the best fit for your research. If you are not sure, read the appropriate sections before making your selection.

[x] Life sciences   [ ] Behavioural & social sciences   [ ] Ecological, evolutionary & environmental sciences

For a reference copy of the document with all sections, see nature.com/documents/nr-reporting-summary-flat.pdf

Life sciences study design

All studies must disclose on these points even when the disclosure is negative.

Sample size	Sample sizes for each experiments are described in the supplemental notes (HiC - Supplementary Note 1, ATAC-seq - Supplementary Note 8). Sample size of at least two biological replicates was chosen

Data exclusions	For the analysis of transcriptomic data, the hemocyte transcriptome was excluded as it contains only very few, very highly expressed genes. Otherwise, no data was excluded.
Replication	Two biological replicates were used for Hi-C and ATAC-seq experiments, all other are described in the Supplementary Note. Both replicates were successfully sequenced.
Randomization	Not applicable. Samples were collected from a batch of developing eggs.
Blinding	Not applicable. Several developmental stages were studied and the analyses studied statistical differences between them.

Reporting for specific materials, systems and methods

We require information from authors about some types of materials, experimental systems and methods used in many studies. Here, indicate whether each material, system or method listed is relevant to your study. If you are not sure if a list item applies to your research, read the appropriate section before selecting a response.

Materials & experimental systems

n/a	Involved in the study
☒	☐ Antibodies
☒	☐ Eukaryotic cell lines
☒	☐ Palaeontology and archaeology
☐	☒ Animals and other organisms
☒	☐ Human research participants
☒	☐ Clinical data
☒	☐ Dual use research of concern

Methods

n/a	Involved in the study
☒	☐ ChIP-seq
☒	☐ Flow cytometry
☒	☐ MRI-based neuroimaging

Animals and other organisms

Policy information about studies involving animals; ARRIVE guidelines recommended for reporting animal research

Laboratory animals	Eggs for Euprymna scolopes were obtained from laboratory reared cultures from the labs of S. Nyholm, J. Foster and the MBL cephalopod initiative. Embryos included both sexes and no specific strains are available for the species. Embryos used in this study had developed between 10 and 22 days.
Wild animals	No wild animals were used for this study.
Field-collected samples	The study did not include samples collected from the field.
Ethics oversight	All animals were treated according to the European Guidelines for Cephalopod care and welfare, i.e., no ethical approval or guidance was required for work done in embryos. Animal collection at MBL was done according to the US regulations.

Note that full information on the approval of the study protocol must also be provided in the manuscript.
